# The evolution of the dystroglycan complex, a major mediator of muscle integrity

**DOI:** 10.1242/bio.012468

**Published:** 2015-08-28

**Authors:** Josephine C. Adams, Andrea Brancaccio

**Affiliations:** 1 School of Biochemistry, University of Bristol, Biomedical Sciences Building, University Walk, Bristol BS8 1TD, UK; 2 Istituto di Chimica del Riconoscimento Molecolare, CNR, Istituto di Biochimica e Biochimica Clinica, Università Cattolica del Sacro Cuore, Largo Francesco Vito 1, Roma 00168, Italy

**Keywords:** Dystroglycan, Protein domain analysis, Metazoa, Multicellularity, Basement membrane, Dystroglycanopathy

## Abstract

Basement membrane (BM) extracellular matrices are crucial for the coordination of different tissue layers. A matrix adhesion receptor that is important for BM function and stability in many mammalian tissues is the dystroglycan (DG) complex. This comprises the non-covalently-associated extracellular α-DG, that interacts with laminin in the BM, and the transmembrane β-DG, that interacts principally with dystrophin to connect to the actin cytoskeleton. Mutations in dystrophin, DG, or several enzymes that glycosylate α-DG underlie severe forms of human muscular dystrophy. Nonwithstanding the pathophysiological importance of the DG complex and its fundamental interest as a non-integrin system of cell-ECM adhesion, the evolution of DG and its interacting proteins is not understood. We analysed the phylogenetic distribution of DG, its proximal binding partners and key processing enzymes in extant metazoan and relevant outgroups. We identify that DG originated after the divergence of ctenophores from porifera and eumetazoa. The C-terminal half of the DG core protein is highly-conserved, yet the N-terminal region, that includes the laminin-binding region, has undergone major lineage-specific divergences. Phylogenetic analysis based on the C-terminal IG2_MAT_NU region identified three distinct clades corresponding to deuterostomes, arthropods, and mollusks/early-diverging metazoans. Whereas the glycosyltransferases that modify α-DG are also present in choanoflagellates, the DG-binding proteins dystrophin and laminin originated at the base of the metazoa, and DG-associated sarcoglycan is restricted to cnidarians and bilaterians. These findings implicate extensive functional diversification of DG within invertebrate lineages and identify the laminin-DG-dystrophin axis as a conserved adhesion system that evolved subsequent to integrin-ECM adhesion, likely to enhance the functional complexity of cell-BM interactions in early metazoans.

## INTRODUCTION

Basement membranes are a specialised and structurally distinct form of metazoan extracellular matrix (ECM). They are composed of a plethora of interacting glycoproteins, predominantly laminin, collagen IV, perlecan and nidogen, that are secreted from surrounding cellular layers and assembled into dense, thin, proteinaceous sheets by cell-ECM and extracellular interactions (Breitkreutz et al., 2013). Basement membranes function to separate epithelial, endothelial, muscle, or neuronal cell layers from other tissue layers and compartments (Halfter et al., 2013). Laminins are amongst the most prominent ECM components of basement membranes and are essential for the assembly of the molecular networks that underlie basement membrane structure (Hohenester and Yurchenco, 2013). Laminin molecules are αβγ heterotrimers and the C-terminal end of the laminin α-subunit is pivotal in the entire network due to its series of laminin globular (LG) domains, which harbour binding sites for multiple cellular receptors including integrins, syndecans, Lutheran blood group glycoprotein and dystroglycan (DG) (Durbeej, 2010). The latter represents the major, non-integrin, laminin receptor in mammals, due to its wide tissue distribution and important pathophysiological roles (Winder, 2001).

**Fig. 1.**
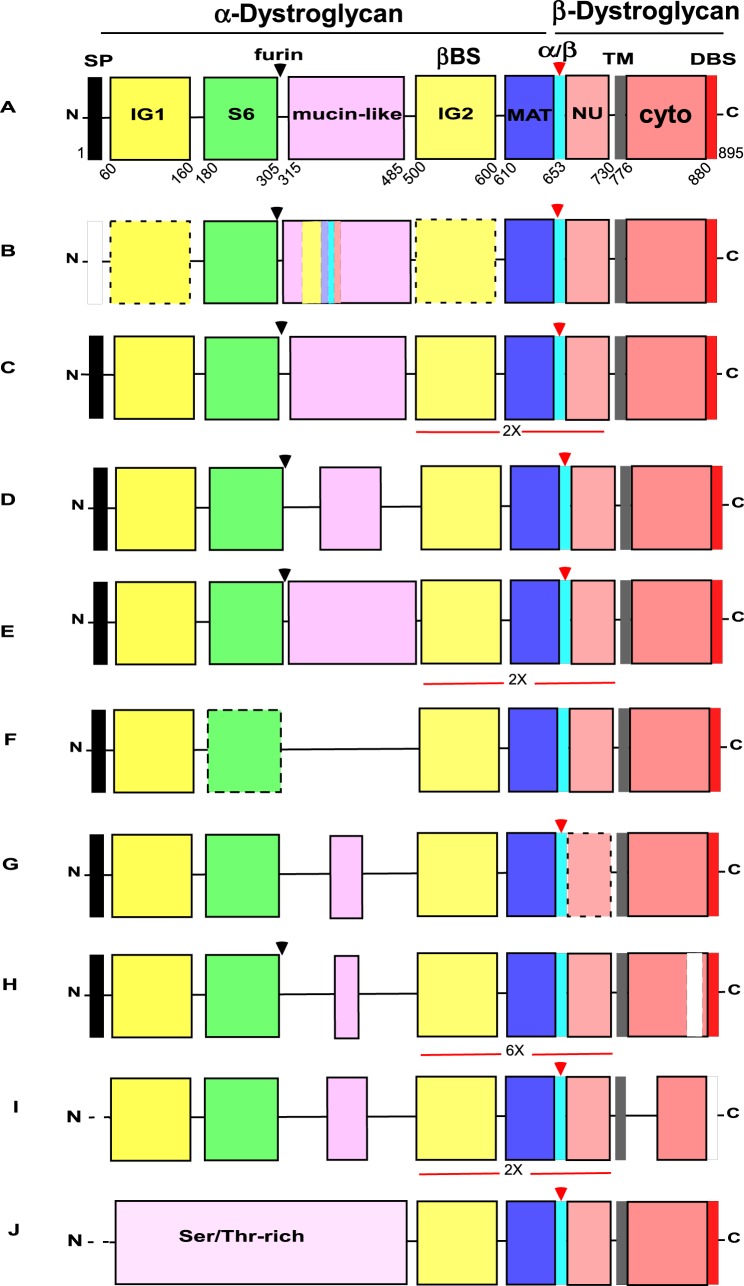
**Domain architectures of dystroglycans from different animal phyla.** (A) The DG typical of vertebrates *Callorhincus milii* (elephant shark), *Lethenteron japonicum* (Cyclostomata), (*Strongylocentrotus purpuratus* (Echinoderma) and an annelid, *Capitella teleta*. (B-J) domain architectures of DG identified in (B) Urochordata (*Ciona intestinalis*) and Cephalochordata (*Branchiostoma lanceolatum*), (C) Hemichordata (*Saccoglossus kowalevskii*), (D) Mollusca, (Gastropods *Lottia gigantea* and *Aplysia californica,* Bivalve, *Crassostrea gigas* and cephalopod, *Octopus vulgaris*), (E) arthropod classes (Insecta and Hymenoptera, *Camponotus floridanus* and others) (see also supplementary material Fig. S1), (F) Nematoda (*Caenorhabditis elegans* and *Caenorhabditis remanei*), (G) Cnidaria, *Hydra magnipapillata*, (H) Cnidaria, *Nematostella vectensis* (see also supplementary material Fig. S2), (I) Placozoa (*Trichoplax adhaerens*), (J) Porifera (homoscleromorph *Oscarella carmela*) (see also supplementary material Fig. S3). Expansions of the IG2_MAT_NU module are indicated in C, E and I (2×) and in H (6×). Black arrowheads indicate the furin cleavage site. Red arrowheads indicate the Gly-Ser α/β maturation site. SP, signal peptide; IG1 and IG2, immunoglobulin-like domains; S6, S6-like domain; βBS, β-subunit binding site on the IG2 domain; MAT, C-terminal region of α-dystroglycan upstream of the Gly-Ser maturation site; NU, natively unfolded region that forms the N-terminal region of the ectodomain of β-dystroglycan; TM, transmembrane; cyto, cytoplasmic domain; DBS, dystrophin-binding site. The SP is reported as a black box if complete, or a white box if partial. Dotted lines indicate protein sequences that are incomplete at the N-terminal end. Dotted boxes around the IG domains of Urochordata (B) or the S6 domain of nematodes (F) indicate the divergence of these domains (less than 20% sequence identity). The dotted box for NU in *H. magnipapillata* DG (G) indicates the presence of two deletions in this region. The white box within the cytoplasmic domain of *N. vectensis* DG (H) indicates the presence of an insertion. In *T. adhaerens* DG (I), no DBS was detected (white box). Diagrams are not to scale. Accession codes and other details are in Table 2.

The mature form of DG is a type I transmembrane protein composed of two non-covalently interacting subunits: α-DG, which is extracellular and highly glycosylated, and β-DG, which contains the transmembrane and cytoplasmic domains (Ibraghimov-Beskrovnaya et al., 1992) (Fig. 1A). The two subunits are produced from a single transcript, with α-DG liberated in the endoplasmic reticulum by an unknown enzyme. During trafficking through the Golgi apparatus, crucial glycosyltransferases, SGK196, an O-mannose kinase (Yoshida-Moriguchi et al., 2013), and B4GAT1, a glucuronyltransferase (Praissman et al., 2014; Willer et al., 2014), act in a concerted and chronologically regulated fashion to modify the α-DG core protein. These modifications are essential for the downstream enzymatic action of like-acetylglucosaminyltransferase **(**LARGE), which adds a repeating disaccharide unit [-α3-GlcA-β3-Xyl-] to a mucin-like, central region of α-DG. These carbohydrate decorations of α-DG are important for the calcium-dependent, high-affinity binding of α-DG to the LG4 and LG5 domains of laminin α-subunits (Barresi and Campbell, 2006; Hara et al., 2011a; Tisi et al., 2000; Harrison et al., 2007) as well as to a number of other LG domain-containing extracellular ligands of α-DG, including agrin, perlecan and neurexin (see Sciandra et al., 2013 and references therein). Intracellularly, the extreme C-terminus of β-DG binds to the cysteine-rich, C-terminal domain of the actin-binding protein, dystrophin, ensuring a connection to the F-actin cytoskeleton (Jung et al., 1995). The C-terminus also interacts with the dystrophin-related protein, utrophin, which is predominantly expressed at neuro-muscular junctions (Ishikawa-Sakurai et al., 2004).

The bridging function of the mature DG complex between external basement membranes and the intracellular F-actin cytoskeleton is considered to be a major determinant of sarcolemma and fibre stability in skeletal muscle (Holmberg and Durbeej, 2013), where the DG complex functions as part of the dystrophin-glycoprotein complex (DGC), a multi-protein complex originally identified in rabbit skeletal muscle (Ervasti and Campbell, 1991; Ibraghimov-Beskrovnaya et al., 1992). The transmembranous core of the complex includes sarcoglycans and the tetraspanin-like protein, sarcospan, as well as DG (Ervasti and Campbell, 1993). It is now appreciated that the DGC has an important role in the mechanical stability of multiple mammalian tissues in addition to skeletal muscle, including the neuromuscular junction, neurons and myelinating Schwann cells (Walko et al., 2013).

DG also has important roles in human pathologies. The abnormal glycosylation of its α-subunit and/or shedding by metalloproteases of its β-subunit ectodomain have been linked to cancer progression (Sgambato and Brancaccio, 2005). α-DG is the receptor for some haemorrhagic fever-causing Arenaviruses and for *Mycobacterium leprae*, the causative agent of leprosy (Cao et al., 1998; Rambukkana et al., 1998). In addition, mutations in proteins responsible for DG glycosylation are causal for a series of severe human neuromuscular disorders including muscle-eye-brain (MEB) disease, Walker-Warburg syndrome (WWS) and limb-girdle muscular dystrophy (LGMD) (Endo, 2015; Graziano et al., 2015). The conditional knock-out of DG in mouse skeletal muscle also leads to severe muscular dystrophy (Cohn et al., 2002). A common molecular scenario behind an enlarging subgroup of DG-related muscular dystrophies, the secondary dystroglycanopathies, is that hypo-glycosylation of DG, originating from genetic abnormalities in the glycosyltransferases that act on DG (Panin and Wells, 2014), leads to reduced binding affinity for laminin-2 in skeletal muscle (Michele et al., 2002). Therefore, glycosylation status also has a crucial role in DG function.

Although DG orthologues have been identified in invertebrate models such as *Drosophila melanogaster* and the nematode *Caenorhabditis elegans* (Greener and Roberts, 2000; Grisoni et al., 2002), many functional and structural aspects of DG remain elusive. Whereas DG has consistent functions in the skeletal muscle and brain of vertebrates, it is much less clear whether DG has the same function(s) in invertebrate animals. Similar to mammals, in *D. melanogaster*, DG is involved in muscle stabilization, the establishment of cellular polarity and important neuronal functions (Shcherbata et al., 2007; Bogdanik et al., 2008; Nakamura et al., 2010; Marrone et al., 2011). In contrast, gene knockout of *DGN-1* in *C. elegans* results not in a muscle phenotype, but in severe disorganization of the somatic gonad epithelium, defects in vulval and excretory cell epithelia, and impaired axon guidance of motor neurons (Johnson et al., 2006; Johnson and Kramer, 2012). Better knowledge of the evolution of DG could improve understanding of the roles of DG in different animals, its physiological roles in mammals, and could reveal novel insights to assist a better understanding of its pathological roles in muscular dystrophies and other human diseases.

Here, we have undertaken a comprehensive study of DG and its proximal associated proteins in order to better understand the evolution of the DG complex and its pathophysiological significance. To our knowledge, this is the first study of the evolution of the DG complex and its associated proteins.

## RESULTS

### Identification of dystroglycans and analysis of conservation of domain architecture

Based on knowledge of vertebrate DGs prior to the start of our study, the domain architecture considered characteristic of a DG, includes, from N-terminus to C-terminus: a signal peptide; immunoglobulin-like domain 1 (IG1); S6 domain (so-called because of its similarity to ribosomal protein S6, Bozic et al., 2004); a mucin-like central region; immunoglobulin-like domain 2 (IG2); the so-called “α/β maturation interface” (MAT) which includes a 50 residue region of α-DG after the IG2 domain and the Gly-Ser site of proteolysis; a natively unfolded domain within the ectodomain of β-DG, (NU); a single transmembrane domain and a cytoplasmic region that includes the dystrophin-binding site (DBS) at its C-terminus (Fig. 1A). To identify DGs across the Metazoa, we first used human dystroglycan as a reference sequence for BLASTP and TBLASTN searches of genomic and transcriptomic databases for species from all the major metazoan phyla and close outgroups for possible DGs. Sequences returned with e-scores <1e−10 were first validated as DGs by reciprocal BLAST searches, by investigation of their domain architectures, and by phylogenetic analyses (the latter are reported in a following section). Newly-identified DGs were also used for further BLAST searches of the early-diverging metazoa. Genome-predicted protein sequences were validated to correspond to transcripts by the identification of corresponding expressed sequence tags (ESTs) and/or cDNA sequences within transcriptome datasets. From this study, DGs were identified only in metazoans. No DGs or DG-like proteins were identified in the unicellular relatives of the Metazoa, the choanoflagellates *Monosiga* *brevicollis* and *Salpingoeca rosetta* or the filasterean, *Capsaspora* *owczarzaki*.

#### Vertebrata and Cyclostomata

DG is conserved throughout vertebrates and all these DGs have very high sequence conservation with human DG (Table 1). The same domain organization was found from mammals to bony fish, in a cartilaginous fish, *Callorhinchus milii,* and a lamprey (Cyclostomata) (Fig. 1A) (Table 2). It is well-recognised that the early bony fish lineage underwent a third round of whole genome duplication (Taylor et al., 2003). We previously demonstrated two paralogues of DG in *Tetraodon nigroviridis,* *Takifugu rubripes*, *Oryzias latipes* and *Gasterosteus aculeatus*, and showed that in *T. nigroviridis* the isoforms are correctly transcribed and spliced out (Pavoni et al., 2007). In the present study, duplicated DG gene products were identified in additional species of fish, namely, the Acanthomorphata *Cynoglossus semilaevis* and *Stegastes partitus*. Duplication of the DG gene was also apparent in the Japanese lamprey, *Lethenteron japonicum* (Table 2).

**Table 1.**
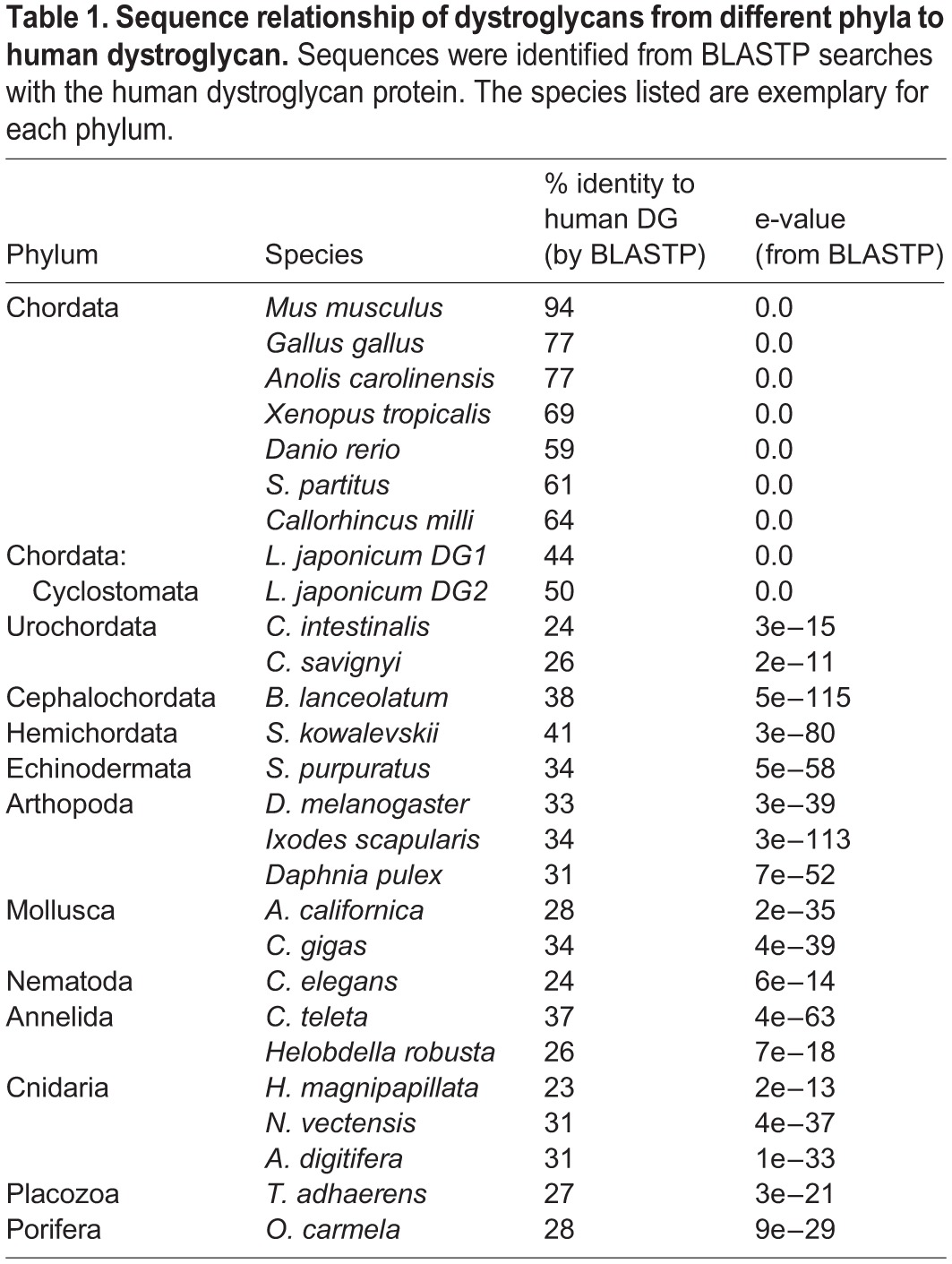
Sequence relationship of dystroglycans from different phyla to human dystroglycan. Sequences were identified from BLASTP searches with the human dystroglycan protein. The species listed are exemplary for each phylum.

**Table 2.**
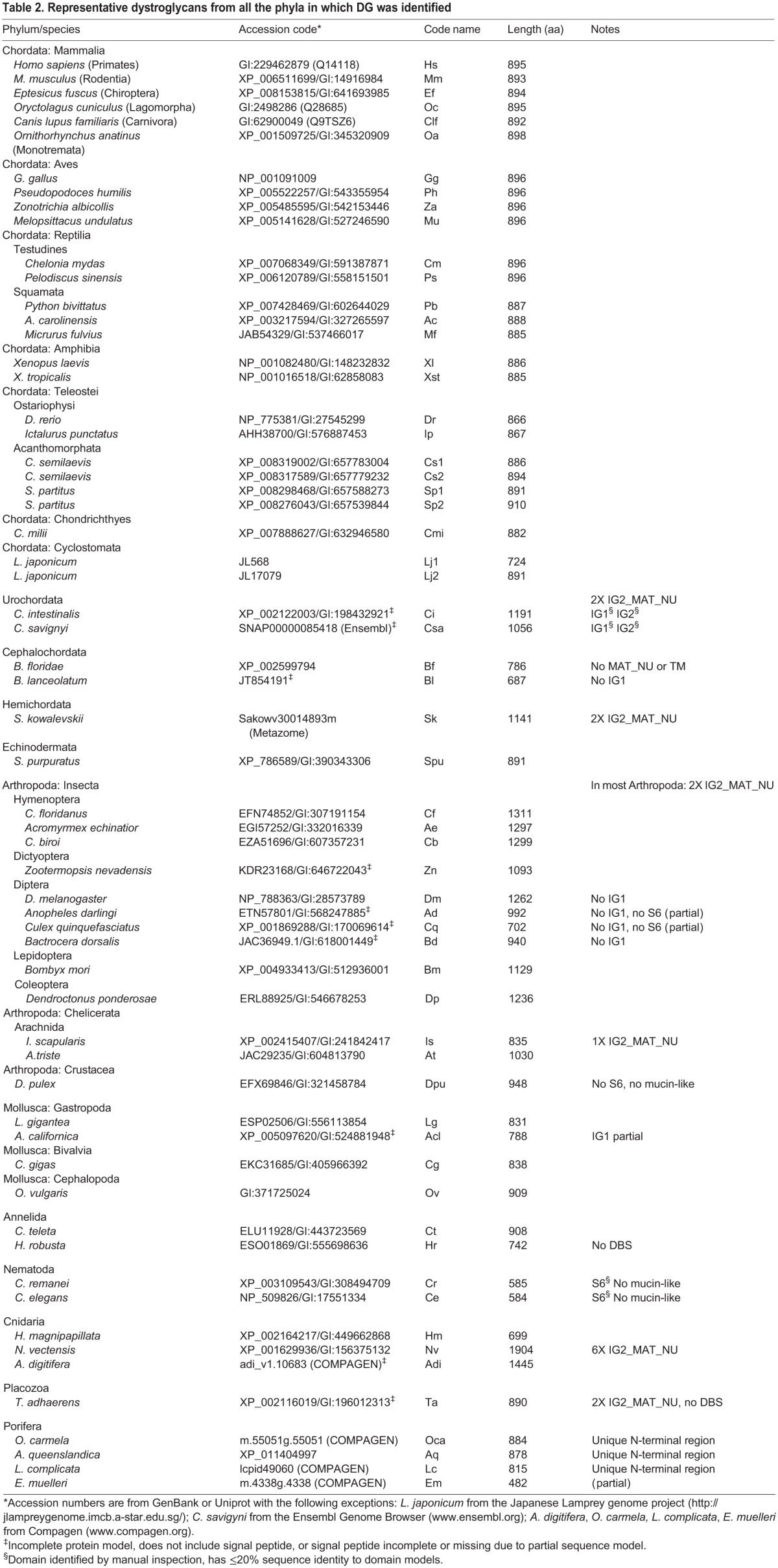
**Representative dystroglycans from all the phyla in which DG was identified**

#### Urochordata, Cephalochordata, Hemichordata, Echinodermata, Mollusca and Annelida

The DGs of Urochordata as represented by *Ciona* *intestinalis* and *Ciona savignyi* are surprisingly weakly related to human DG (Table 1). This is due to extensive sequence divergence and a distinctive domain organization. An insertion of ∼240 aa in the mucin-like region represents a clear repetition of the IG2_MAT_NU module and the two IG domains are very divergent, with <20% sequence identity to a consensus IG domain (Fig. 1B) (Table 2). In contrast, in cephalochordate (*Branchiostoma floridae* and *Branchiostoma* *lanceolatum*) DGs both IG domains as well as the S6 domain are conserved and the domain organization is equivalent to that of vertebrate DGs. The DG of the hemichordate *Saccoglossus* *kowalevskii* is well conserved with vertebrate DGs, yet includes clear repetition of the IG2_MAT_NU module (Fig. 1C). The DG of the echinoderm, *Strongylocentrotus purpuratus,* and DGs from the available species of Mollusca and Annelida all also show high conservation of domains and domains organization with vertebrate DGs (Tables 1 and 2), although the mucin-like region is in all cases shorter than in vertebrate DGs (Fig. 1D) (Table 2). In all these phyla, the most highly conserved region is the C-terminal section of DG, encompassing the IG2_MAT_NU region, the transmembrane and cytoplasmic domains and the dystrophin-binding site.

#### Arthropoda

In many cases, the DGs of Arthropoda are longer (comprising 1000 to 1200 residues) than the DGs of vertebrates (∼900 residues) (Table 2). This is mainly due to a tandem duplication of the IG2_MAT_NU module, which typically spans around 250 aa (Fig. 1E). This property was previously identified for *D. melanogaster* DG (Sciandra et al., 2001). We identify here that this duplication is present in species from all classes of arthropods (insects, crustacea and chelicerates) (Fig. 1E and supplementary material Fig. S1). These data imply that duplication of the IG2_MAT_NU module must have taken place very early in the arthropod lineage. Apart from the IG2_MAT_NU module, the most highly conserved region in the arthropod DGs is the cytoplasmic domain. A striking distinction of the DGs of diptera is the absence of the IG1 domain (supplementary material Fig. S1C). Interestingly, the characterized alternatively-spliced isoforms of *D. melanogaster* DG also include an isoform that lacks a central mucin-like region (Schneider and Baumgartner, 2008). Although the DGs of hymenopteran and dictopteran insects contain the α/β maturation site in the C-terminal IG2_MAT_NU region, this site is not present in DGs from species of Diptera, Lepidoptera or Coleoptera (supplementary material Fig. S1C,F, and see section below).

#### Nematoda

The DGs of nematodes, as represented by *C. elegans* and *Caenorhabditis remanei,* lack a central mucin-like region and thus these are the shortest (<600 aa) DG sequences identified (Table 2). All other domains except the S6 domain are well-conserved (Fig. 1F). None of the available nematode DG sequences contain an α/β maturation site.

#### Cnidaria

**Fig. 2:**
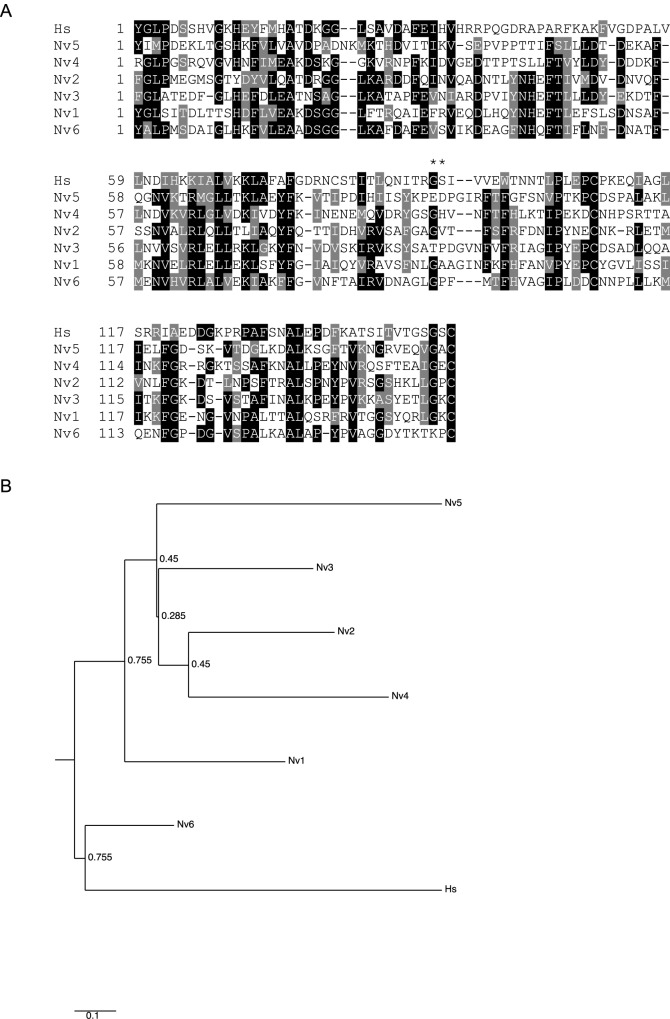
**. Analysis of the multiple repetitions of the IG2_MAT_NU region in *N. vectensis* DG.** (A) Multiple sequence alignment of the six IG2_MAT_NU modules of *N. vectensis* DG compared with this region of human DG. Alignments were prepared in MUSCLE 3.8 and are presented in Boxshade. Asterisks indicate the GS proteolysis site in human DG. At each position, black background includes identical residues; grey background indicates conservative substitutions, and white background indicates non-conserved residues. (B) Phylogenetic analysis of the six IG2_MAT_NU modules of *N. vectensis* DG (alignment of 153 positions) compared with the same region of human DG. The tree was prepared in PhyML with 200 cycles of boot-strapping. Numbers indicate bootstrap support values, with 1 as maximum. Scale=substitutions/site.

Predicted DG proteins were identified in cnidarian species (Tables 1 and 2). The DGs of *Hydra magnipapillata* (Fig. 1G) and *Nematostella vectensis* (Fig. 1H) have unusually short mucin-like regions and the α/β maturation site is not conserved in the latter. *N. vectensis* DG is characterized by extensive duplication of the IG2_MAT_NU region, leading to an unusual domain architecture in which six such modules are present in tandem (Fig. 1H). Each of these modules has a distinct sequence (Fig. 2A, supplementary material Fig. S2). Phylogenetic analysis revealed that the sixth, most C-terminal module is distinct in being the most closely related to human IG2_MAT_NU, whereas each of the other modules are more closely related to each other than to the sixth repeated region (Fig. 2B). *N. vectensis* DG is also characterized by an inserted sequence in the cytoplasmic domain (Fig. 1H).

#### Placozoa

The DG of the placozoan, *Trichoplax* *adhaerens*, includes a duplication of the IG2_MAT_NU module and has a shorter mucin-like region and a cytoplasmic region that lacks the dystrophin-binding site (Fig. 1I, Table 2).

#### Porifera

DG-like proteins were identified in multiple classes of sponges*.* A clear DG-like protein was identified in the homoscleromorph sponge, *Oscarella* *carmela* (Table 1)*.* The most conserved regions are again the IG2_MAT_NU module, the cytoplasmic domain and the C-terminal end of β-DG. The typical N-terminal domain architecture of α-DG and the central mucin-like region are not present, instead the N-terminal half of the protein consists of an extended Ser/Thr-rich region of around 500 aa (Fig. 1J, Table 2). By use of both *O. carmela* and human DG in BLAST searches of genomes or transcriptomes of other sponge species, DG-like proteins with similar domain organisations were identified in the desmosponges *Amphimedon queenslandica* and *Ephydatia* *muelleri* and the calcerous sponge *Leucosolenia* *complicata* (Table 2). Although the N-terminal regions of these proteins are very divergent in sequence, all are Ser/Thr-rich, which is a common characteristic of mucin domains. The C-terminal, DG-like domains are more closely related in sequence (supplementary material Fig. S3).

### Conservation of structurally and functionally important motifs within the α and β dystroglycan subunits

#### Motifs in α-DG

**Fig. 3.**
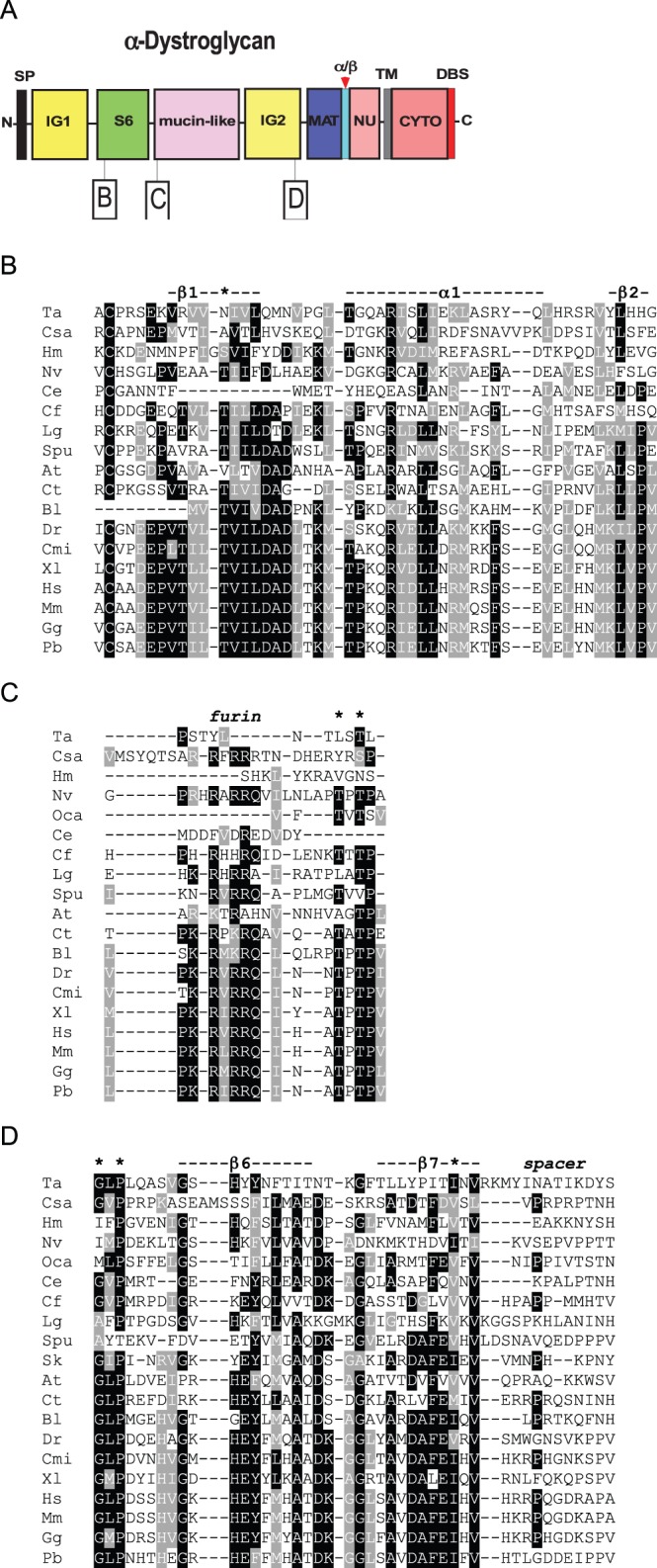
**Multiple sequence alignments of functionally important regions from α-dystroglycan.** The dataset includes representative species from the phyla in which DG was identified. (A) Schematic of α-DG and the regions presented in the alignments. Key as in Fig. 1. (B) Thr192 (*) and the surrounding secondary structure at the beginning of the S6 domain. (C) The furin cleavage site and the Thr-Pro-Thr motif (* *) at the beginning of the mucin-like region. (D) The last two β-strands (dashed line) of the IG2 domain, followed by a spacer region that precedes MAT. The conserved Gly563, Pro565 and Ile593 are also pinpointed by asterisks. Alignments were prepared as in Fig. 2. Codes for species names are as in Table 2.

As introduced above, the domain architecture of αDG includes the N-terminal IG1 and S6 domains, followed by the central mucin-like region and the IG2 domain; the latter anticipates the α/β maturation interface (Fig. 3A). A number of key functional motifs are recognized within mammalian α-DG. We examined the conservation of these motifs by multiple sequence alignments of DGs from species representative of the phyla identified in our phylogenetic survey.

##### Thr192 within the S6 domain

In human α-DG, Thr192 lies within the first β-strand of the S6 domain which has a basket-like structure (Bozic et al., 2004). This β-strand is followed by a long α-helical stretch and a second short β-strand, corresponding to KLVP in mammalian DGs (Fig. 3B). Thr192 is mutated to Met in a patient affected by limb-girdle muscular dystrophy and cognitive impairment (Hara et al., 2011b). In *C. elegans* α-DG*,* the topological equivalent of Thr192, as well as the entire first β-strand of the S6 domain are missing (Fig. 3B). Interestingly, the 192 position in *C. savignyi* (Chordata), *Amblyomma triste* (Arthropoda, Arachnida) or *T. adhaerens* (Placozoa) α-DGs is occupied by Ala, Val or Asn, respectively. These residues that are less bulky than Met and so possibly are more compatible with the structure of the domain.

##### The furin cleavage site and the subsequent Thr pair

The motif R-x-RRQ represents a furin cleavage site that is considered to be responsible for liberation of the N-terminal domain of α-DG (Kanagawa et al., 2004; Singh et al., 2004). The furin site is well conserved in DGs of vertebrates, *Strongylocentrotus purpuratus* and some mollucs (Fig. 3C). In *H. magnipapillata* the consensus is absent (Fig. 3C), however the DGs of *N. vectensis* (Fig. 3C), and the coral *Acropora* *digitifera* (not shown) contain the consensus motif. The furin site is not present in the DGs of *C. elegans* (Nematoda), *T. adhaerens* (Placozoa) or the insect *Camponotus* *floridanus* (Fig. 3C), but is present in another hymenopteran (*Cerapachys biroi*, supplementary material Fig. S1). The following Thr residues that correspond to Thr317 and Thr319 in human DG (asterisks in Fig. 3C), are considered to be the primary sites for post-translational modifications by like-acetylglucosaminyltransferase (LARGE) and are important for the laminin-binding properties of DG (Hara et al., 2011a). The full motif, Thr-Pro-Thr-Pro, is not present in *Lottia* *gigantea* (Molluscs), *A. triste* (Arachnida), *C. elegans* (Nematoda), *Capitella* *teleta* (Annelida), *S. purpuratus* (Echinodermata), *C. floridanus* (Insecta), *T. adhaerens* (Placozoa), *H. magnipapillata* (Cnidaria), *C. savignyi* (Urochordata) or *O. carmela* (Porifera), (Fig. 3C), indicating relatively weak conservation of this site.

##### Ile 595 within the IG2 domain

The two final β-strands (β6 and β7) of the IG2 domain (De Rosa et al., 2011) are shown in Fig. 3D. The initial Gly and Pro residues (asterisks, Fig. 3D) are believed to be important for the interaction of β-DG with α-DG and the maturation of the DG complex (Sciandra et al., 2009). The proline residue is universally conserved and the glycine residues is widely, but not universally, conserved. In human DG, Ile593 is located in the last β-strand (DAFEI/V motif) and is thought to play a crucial role for the folding of the entire IG2 domain and possibly for the overall stability of the DG complex (Gupta et al., 2011; Pirolli et al., 2014). The relevant Ile position is universally occupied by a hydrophobic residue (asterisk, Fig. 3D). In the IG2 domain of the *O. carmela* DG-like protein (Fig. 1J) the DAFEI/V motif is conserved as MTFEV. However, the full DAFEI/V motif itself is not conserved in β-DG from most protostomes or basal metazoa (Fig. 3D). The “spacer” is a typical linker region that precedes the MAT region (Fig. 3A,D).

#### Motifs in β-DG

**Fig. 4:**
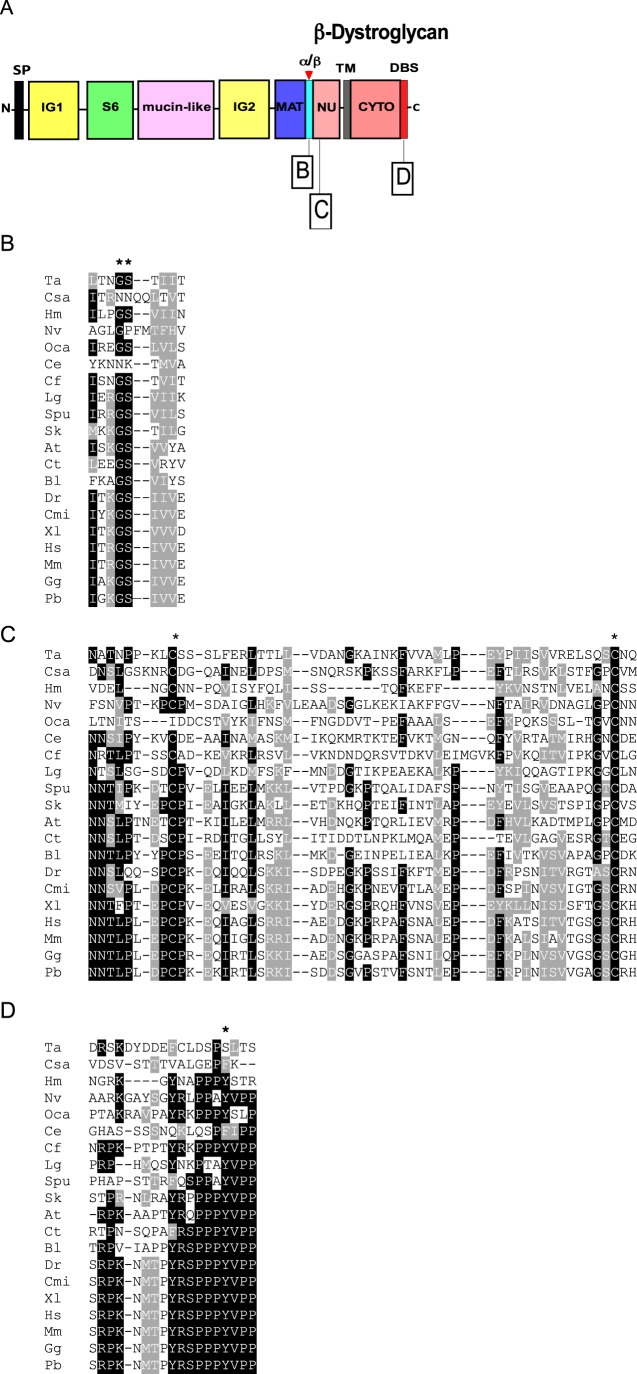
**. Multiple sequence alignments of functionally important regions from β-dystroglycan.** Key as in Fig. 1. (A) Schematic of β-DG and the regions presented in the alignments. (B) The Gly-Ser α/β maturation site (**). (C) Region of the NU domain encompassing the two conserved Cys residues (*). (D) The dystrophin-binding site. The shade coding is as in Fig. 2. Codes for species names are as in Table 2. Tyr892 (*) is a phosphorylation site.

β-DG is liberated upon proteolysis at the α/β cleavage site. It represents the transmembrane subunit of the DG complex and includes the natively unfolded region (Boffi et al., 2001) in its extracellular region and a cytoplasmic domain of about 120 aa that contains the dystrophin-binding site (DBS) at its C-terminus (Fig. 4A).

##### The α/β maturation site

The formation of β-DG involves proteolysis between a Gly and Ser that corresponds to Gly653-Ser654 in human β-DG (Fig. 4A). This event is considered to take place in the endoplasmic reticulum during the maturation and trafficking of the DG complex. The Gly-Ser maturation site is very widely conserved including in the *O. carmela* DG-like protein (Fig. 3B). However, the motif is not conserved in *C. elegans* (as well as in *C. remanei*, not shown), urochordates, several classes of arthropods, and *N. vectensis* β-DG contains a GP motif (Fig. 4B; supplementary material Fig. S1). The GS motif is commonly preceded by a basic residue and followed by a triplet of hydrophobic residues; both are likely to be important for the cleavage event (overall consensus Hy-x-R/K-Gly-Ser-Hy-Hy-Hy. Both these features are well-conserved (Fig. 4B).

##### Cys 669 within the NU domain

Almost the entire NU domain of β-DG is reported in Fig. 4C. The domain includes an interesting pattern of conserved hydrophobic residues that are well conserved with the exception of the DGs of early-diverging metazoans. Two cysteine residues, corresponding to Cys669 and Cys713 in human DG, are thought to form a disulphide bridge within the β-DG ectodomain (Watanabe et al., 2007). Cys 669 was found recently to be mutated to Phe in two siblings affected by a severe form of muscular dystrophy (Geis et al., 2013). Both Cys are highly conserved although a Cys669 equivalent is not present in *O. carmela* DG (Fig. 4C).

##### Y892 and the dystrophin-binding site

The DBS is formed by the last 15 residues of β-DG and binds the cysteine-rich, C-terminal domain of dystrophin (Vulin et al., 2014). The DBS is remarkably conserved in all the phyla apart from Placozoa and urochordates (Fig. 4D). Of note, in *O. carmela* DG-like the relevant C-terminal motif encompassing Y892, YRKPPPY, is fully present although other regions of the cytoplasmic domain are less conserved (Fig. 4D and data not shown). Tyr892 (asterisk, Fig. 4D) has been proposed as a phosphorylation site with a crucial role for the connection of DG to dystrophin or the related protein, utrophin, and for turnover/degradation of the DG complex (James et al., 2000). Interestingly, an Y892F mutation introduced into mice was asymptomatic (Miller et al., 2012) and nematode and urochordate DGs naturally include Phe at this position (Fig. 4D).

### Phylogenetic analysis of dystroglycans

**Fig. 5.**
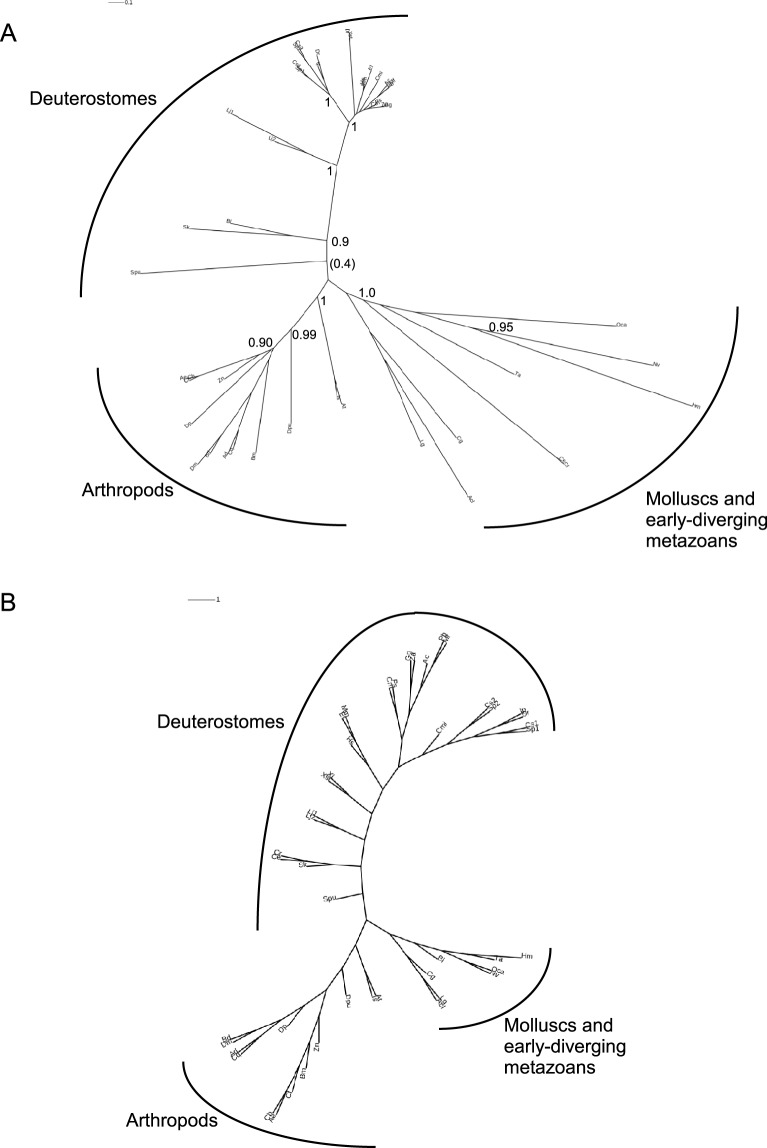
**Phylogenetic analysis based on the IG2_MAT-NU region of dystroglycans.** The IG2_MAT_NU regions from DGs from 46 species (245 positions) were aligned in PRANK and phylogenetic trees constructed (A) in PhyML with 200 cycles of boot-strapping, or (B) as a consensus tree in PROTPARS. Unrooted trees are presented with proportionate branch lengths. Scale bars=substitutions/site. In A, only bootstrap branch support values >0.95 are shown. Codes for species names are as in Table 2.

In view that the IG2-MAT-NU region is the most highly conserved region in all DGs in terms of domain organization, and has high sequence identity across species, this region was chosen for phylogenetic analyses of DGs. Initial analyses showed that urochordate or annelid DGs could not be placed stably due to their extreme sequence divergence from other DGs. In the interest of obtaining the most robustly-supported phylogenetic trees, these were not included in the final dataset. The final alignments were based on 245 positions and 46 species that provide taxon representation of all phyla that have DGs apart from urochordates and annelids. A phylogenetic tree prepared from a PRANK alignment by the maximum likelihood method, PhyML, identified three broad clades, comprising DGs from deuterostomes, arthropods, and other invertebrates, respectively. Of these, the arthropod clade was the most strongly supported as a discrete group and in general the deep branches of the tree received only weak bootstrap support (Fig. 5A). Interestingly, given their similar domain architecture to vertebrate DGs, the DGs from molluscs grouped in clade 3 with the early-diverging metazoans. *O. carmela* DG was reported as most closely related to cnidarian DGs (Fig. 5A). A consensus tree from maximum parsimony analysis, PROTPARS, yielded a similar overall tree topology with three clades that corresponded for the most part to those identified by PhyML. However, this analysis placed the nematode DGs on the same branch as *S. kowalevskii* DG (Fig. 5B). Further tests with different sequence alignment algorithms such as MUSCLE did not yield improvements to this tree topology.

### Origin of the laminin-dystroglycan-dystrophin axis in early-diverging Metazoa

**Fig. 6.**
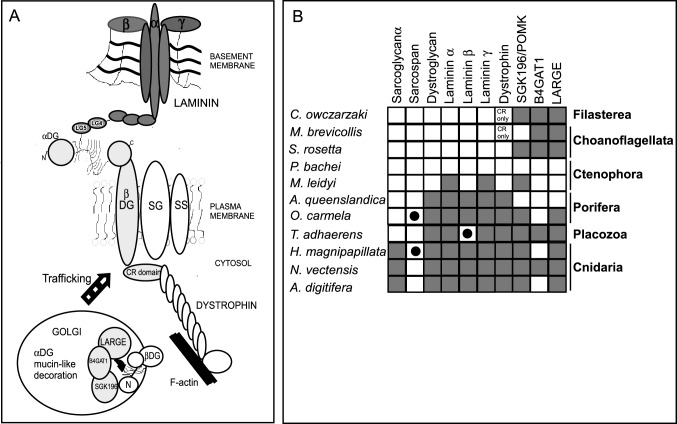
**Evolution of dystroglycan-binding proteins.** (A) Schematic of the interactions of dystroglycan with other members of the DGC and the modifying enzymes that act on DG. In the Golgi complex, α-dystroglycan is post-translationally modified at multiple Thr/Ser residues in its mucin-like region during its trafficking to the cell surface. CR, cysteine-rich domain; SG: sarcoglycan; SS: sarcospan. (B) The phylogenetic distributions of DG, dystroglycan-binding proteins of the DGC and DG-modifying enzymes in early-diverging metazoans and their closest unicellular relatives. Species are representative of the indicated phyla. Key: Grey squares, predicted protein identified, BLASTP e-value<1e−10; black circles, BLASTP e-value>1e−10 and <0.05; white squares, no homologue identified. See supplementary material Table S1 for accession numbers.

As described in the Introduction, DG is the central member of the major non-integrin, laminin-binding, cell-ECM adhesion complex of mammals, the dystrophin-glycoprotein complex. Given the importance of extracellular, membrane-associated and intracellular binding partners of DG for the functionality of the DGC and post-translational processing of DG for its function and binding activities at the plasma membrane (Fig. 6A), we next investigated the phylogenetic distribution of these proteins in comparison to DG itself. Initial studies showed that all these proteins are present in bilaterians, therefore we focused our study on the early-diverging metazoa and certain unicellular eukaryotes, choanoflagellates and the filasterian *C. owczarzaki,* that are the closest outgroups to the metazoa*.* It was important to consider these outgroups because other cell adhesion receptors, integrins and cadherins, evolved before the emergence of metazoans (Abedin and King, 2008; Sebé-Pedrós et al., 2010).

The glycosyltransferases POMK, B4GAT1 and LARGE1 are present in choanoflagellates, *C. owczarzaki* and all the metazoan phyla with the exception of ctenophores (Fig. 6B; supplementary material Table S1). The latter are now considered to be the earliest-diverging lineage of metazoans (Ryan et al., 2013; Moroz et al., 2014). The intracellular binding partner of DG, dystrophin, is conserved in porifera, placozoa and cnidarians. This is of interest given that desmosponges and *T. adhaerens* lack basement membrane ECM structures (reviewed by Adams, 2013). Dystrophin was not identified in ctenophores, or in the unicellular organisms, yet the DG-binding, cysteine-rich domain is present in some otherwise-unrelated proteins of choanoflagellates and *C. owczarzaki* (Fig. 6B; supplementary material Table S1).

With regard to extracellular ligands of α-DG, we restricted our comparative analysis to laminin chains, because this is the best-characterised interaction and because of the predominant role played by the laminin-DG axis in skeletal muscle and other tissues. Orthologues of laminin α, β and γ subunits were present in porifera including *A. queenslandica*, the placazoan *T. adhaerens* and cnidarians (Fig. 6B; Ozbek et al., 2010; Tucker and Adams, 2014). Three predicted proteins most highly related to laminin α or laminin γ subunits were identified in the ctenophore *Mnemiopsis* *leidyi*, but not in *Pleurobrachia bachei*. The *M. leidyi* sequences are very likely incomplete predictions as no signal peptides were identified (supplementary material Table S1). As previously reported, no secreted laminin proteins were identified in the choanoflagellates or *C. owczarzaki* (Williams et al., 2014). These results confirm laminins as a metazoan innovation (Fig. 6B).

In common with DG itself, sarcospan, the transmembrane protein of the dystrophin-glycoprotein complex, appears to have evolved after the divergence of ctenophores. Sarcospan was identified in *O. carmela*, but not in *A. queenslandica*, *L. complicata* or *E. muelleri*. Loss of the sarcospan gene appears relatively common, as sarcospan was not identified in *T. adhaerens* and was present only in *H. magnipapillata* out of the three cnidarian species examined (Fig. 6B). The other transmembrane protein of the dystrophin-glycoprotein complex, sarcoglycan, was identified in cnidarians but not in other basal metazoa, and thus is inferred to have a later evolutionary origin that the other components (Fig. 6B).

## DISCUSSION

The studies reported here reveal that dystroglycan and its associated proteins are widely conserved within the metazoa. However, unlike the integrin and cadherin cell adhesion receptors and their major intracellular binding partners (Abedin and King, 2008; Sebé-Pedrós et al., 2010), dystroglycan and dystrophin were not identified in any unicellular organisms, nor in the earliest-branching metazoans, the ctenophores. These findings lead to the model that DG and the DGC evolved subsequent to the divergence of the ctenophores, and thus within the context of a multi-cellular metazoan ancestor in which an ECM with laminin-integrin adhesion and signaling was already active. This evolution might have been driven by selection for enhancement of the stability and/or functional repertoire of cell interactions with basement membranes. Another outcome of our analysis of the domain architecture and sequences of DGs from different metazoan phyla is the demonstration of considerable, lineage-specific divergence of DG proteins in invertebrates. This unexpected finding provides a new context for considering the functions of DG in muscle and other tissues.

### Genesis of the domain architecture of dystroglycan

Our molecular phylogenetic study of DG establishes that the most highly conserved region of DG is the IG2_MAT_NU region, the transmembrane domain and the cytoplasmic domain that includes the C-terminal dystrophin-binding site (DBS). These domains are present in DGs from all phyla in which DGs were identified. Since these domains include the β-DG binding site, the α/β processing site and the transmembrane domain, the region is likely under strong, uniform selection for correct retention and presentation of DG at the plasma membrane. Direct functional evidence for the importance of the cytoplasmic domain of DG in non-muscle tissues include the establishment of oocyte polarity in *D. melanogaster* (Deng et al., 2003) and visual impairment in mice due to *knockin* in the retina of a form of DG lacking the entire cytoplasmic domain (Satz et al., 2009). Of the functional motifs within this region, the most universally conserved is the DBS (Figs 3 and 4). In addition to dystrophin binding, the DBS is thought to act as a binding platform for additional interacting proteins, being possibly involved in the regulation of signalling pathways (Huang et al., 2000; Moore and Winder, 2010). Because the cysteine-rich domain that contains the β-DG binding site of dystrophin (Vulin et al., 2014), clearly evolved prior to the origin of the Metazoa (Fig. 5B), it is possible that an interaction of the cysteine-rich domain and DBS (in the context of otherwise unrelated proteins) predates the origin of DG and dystrophin. However, we did not identify protein sequences related to the cytoplasmic domain of DG in choanoflagellates.

In contrast, the N-terminal region, comprising the IG1 domain, S6 domain, and mucin-like region shows much greater variability, including extreme sequence divergence of the IG1 or S6 domains, great variability in the length of the mucin-like region, or complete loss of the S6 or mucin-like regions (Fig. 1). This variation is intriguing given the central importance ascribed to laminin-binding by DG for its function as a cell-ECM adhesion receptor. The N-terminus, comprising IG1 and S6 domains, is involved in the maturation/glycosylation pathway of α-DG and is thought to represent a targeting site for the Golgi glycosyltransferases that act on residues within the mucin-like region (Kanagawa et al., 2004; Singh et al., 2004; Panin and Wells, 2014). Furthermore, early studies of α-DG revealed this N-terminal region to represent an autonomously-folding protein domain (Brancaccio et al., 1995, 1997). In view that the furin site that liberates the N-terminal domain in the Golgi during post-translational maturation of α-DG is conserved in many phyla, the N-terminus of α-DG may have important yet undiscovered separate functions in cells and tissues (Hesse et al., 2011). The identification of this fragment of DG in human cerebrospinal fluid (Saito et al., 2011) also supports the possibility of extracellular roles separate from the DG complex. These considerations indicate that the IG1 and S6 domains could indeed be under selection pressures distinct from those acting on other regions of DG.

The variability of the mucin-like region is of particular interest in view of the central role of this region in laminin-binding and also with regard to the general role of mucins that can sterically affect integrin clustering (Paszek et al., 2014). Whether DGs that lack this region, for example the DGs of nematodes, have laminin-binding capacity will require direct experimental tests. The IG1 domain binds weakly to laminin and could provide an alternative mode of interaction (Bozic et al., 2004). Similarly, the pair of threonines in α-DG that are modified by LARGE are not conserved in multiple phyla (Fig. 3). It is possible that other threonine residues within the mucin-like region could be LARGE targets in these lineages, or that binding to other ECM ligands such as perlecan has evolved to predominate over laminin-binding.

We identified that duplication events involving the IG2_MAT_NU module have occurred in multiple phyla. Tandem duplication of the IG2_MAT_NU module was observed in *S. kowalewskii*, all Arthropoda and *T. adhaerens* (Placozoa), and a six-fold repetition in *N. vectensis* DG. We speculate that these enlargements of the DG molecule may have conferred evolutionary advantages by imposing a more rigid structure of the DG extracellular region and the presentation of the N-terminal domain and laminin-binding region at a greater distance from the cell-surface. A curious related observation is the extreme sequence divergence of the *Ciona* DGs from all other DGs, even within the IG2-MAT-NU region. Uniquely, in the DGs of urochordates, a repeated IG-MAT-NU region is present as an insertion within the mucin-like region. Because the LARGE modification site is missing (Fig. 3C) it is unclear if these DGs can function as laminin-binding proteins. For comparison, *Ciona* laminin subunits and dystrophin have around 40% sequence identity to the human orthologues (BLASTP search results). Thus, the extreme sequence divergence of urochordate DGs is not typical for other proteins within the DGC. Neither sarcospan nor sarcoglycan are encoded in the currently available urochodate genomes. It is possible that DG in modern urochordates may have evolved a distinct function that does not depend on the DGC.

Phylogenetic trees constructed on the basis of the highly-conserved IG2_MAT-NU region returned a consistent tree topology, with division of the DGs of arthropods, deuterostomes, and other invertebrates into three separate clades. Nevertheless, within the deuterostome clade, it is notable that the DGs of invertebrate deuterostomes branch separately and are clearly distinct from the vertebrate DGs. We speculate that DGs may have functional distinctions in these lineages. The arthropod clade was strongly supported, with the caveat that the taxon representation of non-arthropod protostomes and early-diverging metazoans is more patchy than for the arthropods. The clade comprising molluscs and early-diverging metazoans was the least stable under different methods of phylogenetic analysis and contained relatively divergent sequences. In view of the moderate or low level of overall sequence identity between these DGs and others (e.g. Table 1) and the relatively short (245 positions) sequences used for phylogenetic analysis it is perhaps not surprising that bootstrap support for this clade was low. Overall, even though the majority of species have retained a single form of DG in their genomes, it is apparent that there has been extensive sequence divergence even in this most conserved region of DG during the evolution of extant metazoans.

In view of the many examples of divergence of DG domain organisation that we identified in invertebrate DGs, the consistency of domain architecture amongst vertebrates becomes a point of note. We suggest that there have been several contributory factors: first, in vertebrates, the major function of DG as a component of the DGC may have resulted in a strong selection pressure to conserve the domain architecture and post-translational modifications. Indeed, relatively few disease-causing mutations of DG have been identified and these are all point mutations rather than domain deletions. Secondly, vertebrate DG genes have an unusual gene organization in which all of the protein except for the N-terminus is encoded in a single very large exon (Ibraghimov-Beskrovnaya et al., 1993; Leeb et al., 2000). This may limit the possibilities for domain shuffling that can maintain an appropriate open reading frame. An important outcome of our analysis is the identification in multiple species of domain duplications and of forms of DG predicted to be uncleaved; these findings open up new possibilities for comparison of functional properties by expressing these cDNAs in specific cell systems.

### Model for the evolution of the dystrophin-glycoprotein complex

**Fig. 7.**
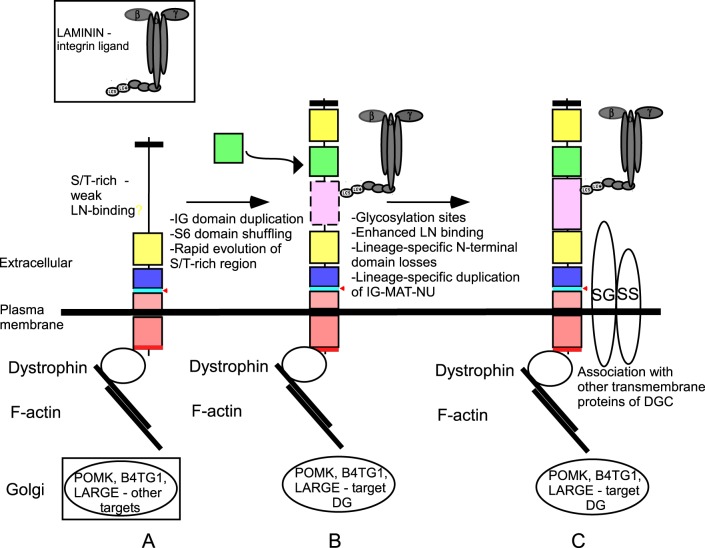
**A model for the evolution of dystroglycan and the DGC.** See text for details.

We propose that the ancestral DG included a signal peptide, the IG2-MAT-NU region, transmembrane and a cytoplasmic domain with a dystrophin-binding motif. This protein might have included a serine/threonine-rich region proximal to the N-terminus; alternatively, this region may have become incorporated subsequently by domain shuffling. We speculate that this protein had weak laminin-binding activity (Fig. 7A). Rapid evolution of the N-terminal region resulting from domain duplication and shuffling of the IG domain then gave rise to the IG1 domain, in conjunction with incorporation of the S6 domain by domain shuffling from another genomic locus, and evolution of the Ser/Thr-rich region to a mucin-like nature with increased laminin-binding activity (Fig. 7B). We propose that these events set the scene for assembly of the DGC complex, by incorporation of *cis*-acting, transmembrane partners of DG and subsequent lineage-specific divergence of DGs in certain phyla (Fig. 7C).

Our findings on the phylogenetic distribution of important DG-binding-proteins are consistent with the model that the DG-binding proteins of the DGC arose within metazoans, with the cytoplasmic dystrophin being of earliest origin (Fig. 7). The presence of laminin subunits throughout extant Porifera, the laminin-like proteins of the ctenophore *M. leidyi,* and the morphologically distinct basement membrane structures of homoscleromorph sponges such as *O. carmela* (Boute et al., 1996) all indicate an earlier evolutionary origin of laminins than DG itself. In the absence of DG, integrins and potentially syndecan (Chakravarti and Adams, 2006), could have functioned as the major laminin-binding cell adhesion receptors. As discussed above, several post-translational modifications of the mucin-like region of DG are important for high-affinity laminin-binding by the DGs of modern animals. The three glycosyltransferases that mediate these modifications all predate metazoans in their evolutionary origins (Fig. 5B). It follows that these enzymes must have different molecular targets within the unicellular organisms and that the targeting of DG by these enzymes must have evolved as a later innovation within the metazoa. It is also notable that B4GAT1 is not always encoded in early-diverging metazoans that have DG: for example, *O. carmela* and several cnidarians lack a B4GAT1 orthologue (Fig. 5). This suggests that glycosylation of the mucin-like region by this enzyme is not obligatory for DG function. Nevertheless, since LARGE is conserved throughout the metazoa and candidate threonine residues for modification by LARGE were identified even in the divergent N-terminal region of *O. carmela,* laminin-binding is viewed as an early-evolved property of DG (Fig. 7).

Because the DG-binding, transmembrane component of the DGC, sarcoglycan, is present only in cnidarians and bilaterians, the most parsimonious model is that sarcoglycan is of a later evolutionary origin than DG (Fig. 7C). However, the alternative possibility of lineage-specific gene loss in porifera and placozoa cannot be ruled out at this time. In contrast, the associated transmembrane protein, sarcospan, appears to have evolved subsequent to the divergence of ctenophores. Although sarcospan is proposed to have important roles in modulating the stability of other components of the DGC, the absence of a sarcospan from *T. adhaerens* and multiple cnidarian species, as well as its absence in *Drosophila* (Greener and Roberts, 2000), indicates that it has been dispensable in many invertebrates. Our model proposes that a dystrophin-DG-laminin axis was functional in metazoans prior to the emergence of the dystrophin-glycoprotein complex (Fig. 7). The diversity of DG sequences identified in invertebrates may thus reflect a wide evolutionary radiation of this axis rather than evolution under selection in the specific context of the DGC.

### Muscle: with or without dystroglycan?

A major question is to understand whether DG and its related proteins have been important for the evolution of striated muscle. Based on recent genomic data, it is now considered that muscle originated independently in ctenophores, the most ancient multicellular animals that display individual muscle fibres (Hernandez-Nicaise et al., 1982, Dayraud et al., 2012). Striated muscle also emerged independently in bilaterians and cnidarians (Steinmetz et al., 2012). We did not identify any DG, dystrophin, sarcoglycan or sarcospan orthologues in ctenophores (Fig. 5), leading to the conclusion that DG and the DGC evolved after the divergence of ctenophores. Overall, this indicates that a form of striated muscle can exist without dystroglycan and the DGC. Indeed, in vertebrates, DG is not strictly essential for muscle differentiation, or for the localization of dystrophin and other components of the DGC to the sarcolemma (Côté et al., 1999, 2002). Its major role is to afford stability and resilience to adult sarcolemma and muscle fibres (Cohn et al., 2002). In *C. elegans*, DG lacks the mucin-like region and does not seem to have an important role either for muscle stability or dystrophin binding (Johnson et al., 2006), although it is possible that a weak laminin-binding activity is provided by the IG1 domain (Bozic et al., 2004). Our molecular phylogenetic data demonstrate that the mucin-like region of DG evolved subsequently to the IG2_MAT_NU core region, thus leading to dramatic changes in the maturation process as well as the domain organization of the entire protein (Fig. 7). The evolution of an increased affinity towards extracellular laminins may have paralleled a substantial increase in the possible strength of musculature in some invertebrate phyla, thus, for example, enabling more complicated and faster movements or higher force generation for feeding or adherence to surfaces.

## MATERIALS AND METHODS

### Identification of dystroglycan and dystroglycan-associated proteins throughout the metazoa

The human DG protein sequence (GI:229462879) was used in systematic BLASTP or TBLASTX searches of NCBI GenBank according to entrez-specified terms for each of the major phyla of metazoans, or species of choanoflagellates and filasterians. Databases of specific genome projects were also searched, including the Japanese Lamprey Genome Project (http://jlampreygenome.imcb.a-star.edu.sg/) (Mehta et al., 2013); the database Metazome v3.0 from University of California (http://www.metazome.net); the *Mnemiopsis* Genome Project Portal (http://research.nhgri.nih.gov/mnemiopsis/) (Ryan et al., 2013); the *Pleurobrachia* Genome (http://neurobase.rc.ufl.edu/pleurobrachia) (Moroz et al., 2014); the platform COMPAGEN at Kiel University (http://compagen.zoologie.uni-kiel.de/index.html) (Hemmrich and Bosch, 2008) for *Oscarella carmela, Leucosolenia complicata*, *Ephydatia muelleri* (Porifera) and the stony coral *Acropora digitifera* (Cnidaria); the JGI Trichoplax Genome Page (http://genome.jgi-psf.org/Triad1/Triad1.home.html) (Srivastava et al., 2008); *Ciona savignyi* CSAV 2.0 resources at ENSEMBL (www.ensembl.org/Ciona_savignyi/Info/Index), and the Broad Institute Initiative on Multicellularity (Suga et al., 2013). Sequences hits returned with scores <1e−10 were validated as dystroglycan orthologues by reciprocal BLASTP searches against the entire GenPeptide database. In many cases, annotated sequences corresponding to DG orthologues were already available, especially for vertebrates which in general show >90% identity with the human sequence. Additional BLAST searches were carried out with DG sequences from early-diverging metazoans, *C. elegans* and *D. melanogaster* for additional identification of DG sequences divergent from vertebrate DGs. Genome-predicted proteins were validated as transcribed sequences by identification of corresponding expressed sequence tags (ESTs) by TBLASTX searches of dbest or the transcriptomic resources at NCBI or the above-mentioned genome databases. From the many DG sequences identified, a curated dataset of 58 sequences from species representative of the major phyla and classes was used for further phylogenetic analyses. DG sequences from parasitic animals: *Schistosoma mansoni* (Platyhelminthes), *Trichinella spiralis* and *Loa loa* (Nematodes), showed extreme sequence divergence and were excluded from this set. The curated dataset of DG sequences is listed in Table 2.

A similar approach, with the respective human protein sequences as the search queries, was taken to identify dystrophin (P11532), laminin-α2 (P24043), laminin-β1 (AAI13456), laminin-γ1 (NP_002284), sarcospan (Q14714), sarcoglycan-alpha (Q16586) and relevant glycosyltransferases (SGK196, Q9H5K3; B4GAT1, O43505 and LARGE1, O95461) homologues in early-diverging metazoan species, choanoflagellates and the filasterean, *C. owczarzaki*. The dataset is listed in supplementary material Table S1.

### Analysis of protein domains and motifs

Protein domain architectures were analysed in InterProScan 5.0 http://www.ebi.ac.uk/Tools/pfa/iprscan5/ (Zdobnov and Apweiler, 2001). DG sequences were examined for the presence of a signal peptide and a transmembrane domain via SignalP 4.1 (Petersen et al., 2011) and TMHMM (Sonnhammer et al., 1998) at http://www.cbs.dtu.dk/services.

### Multiple sequence alignment and phylogenetic analysis

Multiple sequence alignments were performed in MUSCLE 3.8 (Edgar, 2004) or PRANK (Löytynoja and Goldman, 2010) via the resources of EMBL/EBI (http://www.ebi.ac.uk/Tools/msa). Illustrations of multiple sequence alignments are presented in BoxShade 3.21 (http://www.ch.embnet.org/software/BOX_form.html). Phylogenetic trees were prepared from PRANK alignments of the IG2_MAT-NU region (245 positions) from DGs from 46 species representative of all phyla except urochordates and annelids. Trees were prepared in PhyML 3.0 (Guindon et al., 2010) at default parameters through the resources of phylogeny.fr (Dereeper et al., 2008) with 200 cycles of boot-strapping or as a consensus tree in PROTPARS [PHYLIP (Phylogeny Inference Package) version 3.5c.; J. Felsenstein, Department of Genetics, University of Washington, Seattle, USA], at default parameters through the resources of Phylemon 2.0 (Sánchez et al., 2011). Trees were visualized via Interactive Tree Of Life (http://itol.embl.de/) (Letunic and Bork, 2007).

## Supplementary Material

Supplementary information
