# Supplementary material for: The evolution of the dystroglycan complex, a major mediator of muscle integrity
Source: Biol Open. 2015 Aug 28;4(9):1163–79. doi: 10.1242/bio.012468 (PMC4582122; doi:10.1242/bio.012468)
Supplement: Supplementary information [file supp_4_9_1163__index.html]

The evolution of the dystroglycan complex, a major mediator of muscle integrity — Supplementary information 

# The evolution of the dystroglycan complex, a major mediator of muscle integrity

## BIO012468 Supplementary information

**Files in this Data Supplement:**

- Supplementary information
