# Supplementary material for: The evolution of the dystroglycan complex, a major mediator of muscle integrity
Source: Biol Open. 2015 Aug 28;4(9):1163–79. doi: 10.1242/bio.012468 (PMC4582122; doi:10.1242/bio.012468)
Supplement: Supplementary information [file supp_bio.012468_BIO012468supp.pdf]

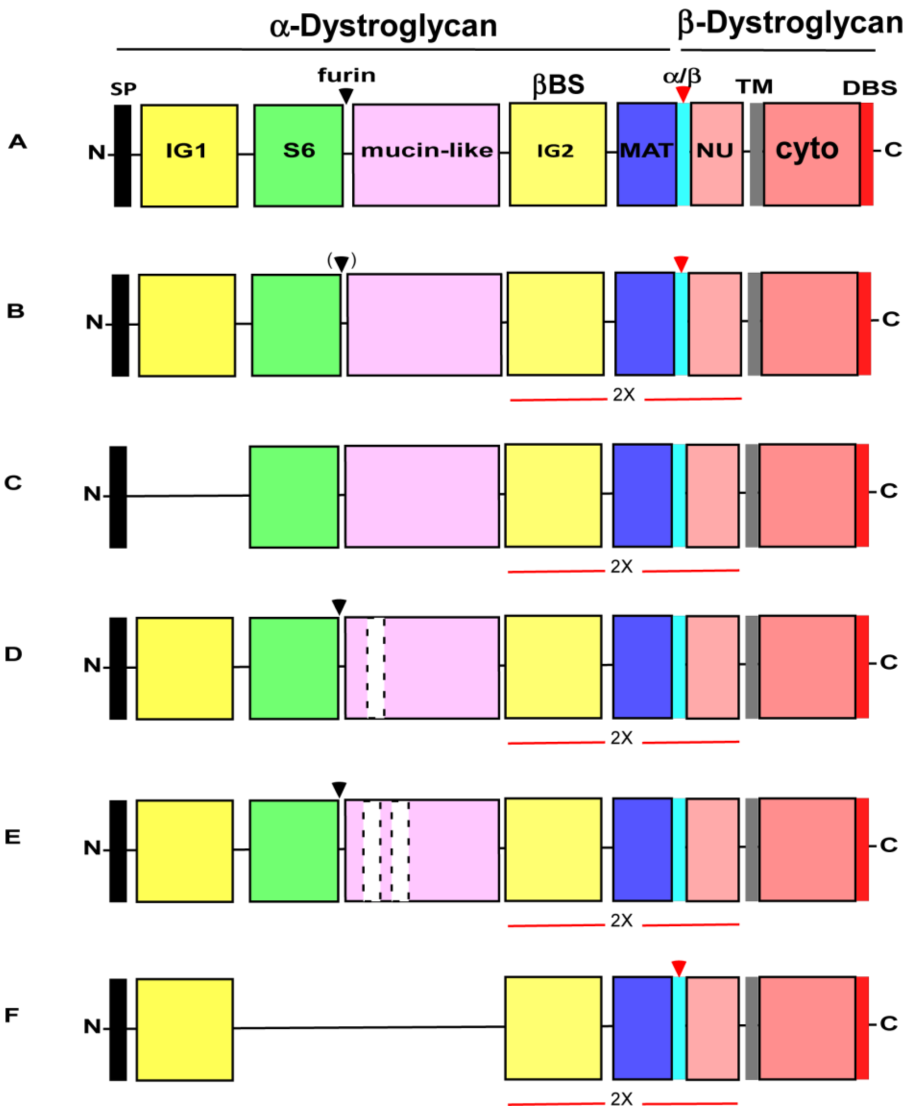

Fig. S1: Domain architectures of dystroglycans from different classes of arthropods.
(A), The typical DG of vertebrates. (B), the domain architecture of most DGs of arthropods (as shown in Fig. 1). B, The DGs of hymenopteran (*Camponotus floridanus, Acromyrmex echinatior, Cerapachys biroi*), dictyopteran insects (*Zootermopsis nevadensis*) and chelicerates (*Amblyomma triste*) have a similar organization to that of vertebrate DGs, and an α/β maturation site. Variant domain organisations are shown in C-F. (C), The DGs of dipteran insects (*Drosophila melanogaster* and *Bactrocera dorsalis*) lack an IG1 domain. (D), The DG of a lepidopteran insect, *Bombyx mori,* has a unique insertion in the mucin-like region. (E), the DG of a coleopteran insect (*Dendroctonus ponderosae*) has two insertions in the mucin-like region. (F), The DG of the crustacean *Daphnia pulex* lacks a mucin-like module but contains a Gly-Ser maturation site. Black arrowheads indicate the furin cleavage site (brackets indicate that this motif is variably present in the DGs represented in (B)). Red arrowheads indicate the conserved α/β maturation site. Key: SP, signal peptide; IG1 and IG2, immunoglobulin-like domains; S6, S6-like domain; βBS, β-DG binding site; MAT, C-terminal region of α-dystroglycan upstream of the maturation site;
NU, natively unfolded region; TM, transmembrane; Cyto, cytoplasmic domain; DBS, dystrophin-binding site.
A white box with dotted lines indicates the presence of an inserted sequence.

Biology Open • Supplementary information

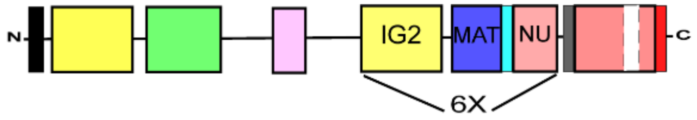


```
                                               1X-------->
Nv_6 copies of IG2_MAT_N              NNAPVVISRLSRVTVYSGQLIRFLIPENTFSDREDGGTRRLNLKLLTLTGATIIPSSWIL
Hs_IG2_MAT_NU_6TIMES                  NQRPELKNHIDRVDAWVGTYFEVKIPSDTFYDHEDTTTDKLKLTLKLREQQLVGEKSWVQ
                                      *: * : ..:.** .: *  : . **.:** *.**  * .*:*.*        :  .**:

Nv_6 copies of IG2_MAT_NU             LNPASQEIYGLSITDLTTSHDFLVEAKDSGGLFTRQAIEFRVEQDLH--QYNHEFTLEFS
Hs_IG2_MAT_NU_6TIMES                  FNSNSQLMYGLPDSSHVGKHEYFMHATDKGGLSAVDAFEIHVHRRPQGDRAPARFKAKF-
                                      :*. ** :***. :. . .*:::: *.*.*** : :*:*:.* .  :  .    *. :*

Nv_6 copies of IG2_MAT_NU             LSDNSAFMKNVELRLELLEKLSFYFGIAIQYVRAVSF-NLGAAGINFKFHFANVPYEPCY
Hs_IG2_MAT_NU_6TIMES                  VGDPALVLNDIHKKIALVKKLAFAFG--DRNCSTITLQNITRGSIVVEWTNNTLPLEPCP
                                      :.* : .::::  .: *::**:* **    .   :::: *:  ..* .::    .:* ***
                                                                                          2X----
Nv_6 copies of IG2_MAT_NU             GVLISSIIKKFGENG--VNPALTTALQSRFRVTGGSYQRLGKCINSRPVVDQGVSSISAI
Hs_IG2_MAT_NU_6TIMES                  KEQIAGLSRRIAEDDGKPRPAFSNALEPDFKATSITVTGSGSC-NQRPELKNHIDRVDAW
                                         *:.: ..:.*:.   .**::.**:. *..*. :    *.* *.** :.: :. :.*
                                      ---->
Nv_6 copies of IG2_MAT_NU             TGQALSYKVPSNAFRDAEDGPSD-LTLNMLTSGLAKLPAMSWVLFNQQTNTIFGLPMEGM
Hs_IG2_MAT_NU_6TIMES                  VGTYFEVKIPSDTFYDHEDTTTDKLKLTLKLREQQLVGEKSWVQFNSNSQLMYGLPDSSH
                                      .*  :. *:**::* * ** .:* *.*.:        :   *** **.::: ::*** ..

Nv_6 copies of IG2_MAT_NU             SGTYDYVLQATDRGGLKARDDFQINV--QADNTLYNHEFTIVMDVDNVQFSSNVALRLQL
Hs_IG2_MAT_NU_6TIMES                  VGKHEYFMHATDKGGLSAVDAFEIHVHRRPQGDRAPARFKAKFVGDPALVLNDIHKKIAL
                                       *.::*.::***.***.* * *:*:*  ..:.       *.  :  * . . .::  .: *

Nv_6 copies of IG2_MAT_NU             LTLIAQYF-QTTIDHVRVSAFGAG-VTFSFRFDNIPYNECNK-RLETMVNLFGKD--TLN
Hs_IG2_MAT_NU_6TIMES                  VKKLAFAFGDRNCSTITLQNITRGSIVVEWTNNTLPLEPCPKEQIAGLSRRIAEDDGKPR
                                      :. :*  * : . . : :. :  * :...:  :.:* : * * .:  : . :.:*  . .
                                                                             3X-------->
Nv_6 copies of +IG2_MAT_NU            PSFTRALSPNYPVRSGSHKLLGPCNKPDSRPPVVGAPIPAIDVFSGQSIMRDIAPNTFKD
Hs_IG2_MAT_NU_6TIMES                  PAFSNALEPDFKATSITVTGSGSCNQ----RPELKNHIDRVDAWVGTYFEVKIPSDTFYD
                                      *:*:.**.*:: . * : .  *.**:     * :   *  :*.: *  :  .*..:** *

Nv_6 copies of IG2_MAT_NU             GIDGDASNLKLDLLARGGIRLSPNSWIQLNGPALQIFGLA-TEDFGLHEFDLEATNSAGL
Hs_IG2_MAT_NU_6TIMES                  HEDTTTDKLKLTLKLREQQLVGEKSWVQFNSNSQLMYGLPDSSHVGKHEYFMHATDKGGL
                                        *  :.:*** *  *    :. :**:*:*. :  ::**. :. .* **: : **:..**

Nv_6 copies of IG2_MAT_NU             KATAPFEVNI------ARDPVIYNHEFTLLLDYEKDTFLNVVSVRLELLRKLGKYF-NVD
Hs_IG2_MAT_NU_6TIMES                  SAVDAFEIHVHRRPQGDRAPARFKAKFV----GDPALVLNDIHKKIALVKKLAFAFGDRN
                                      .*. .**:::        * *. :: :*.      :   .** :  .: *:.**.  * : :

Nv_6 copies of IG2_MAT_NU             VSKIRVKSYSATPDGVNFVFRIAGIPYEPCDSADLQQAITKFGKDS--VSTAFINALKPE
Hs_IG2_MAT_NU_6TIMES                  CSTITLQ--NITRGSIVVEWTNNTLPLEPCPKEQIAGLSRRIAEDDGKPRPAFSNALEPD
                                       *.* ::  . * ..: . :     :* *** . ::      .:.:*.     .** ***:*:
                                                                   4X-------->
Nv_6 copies of IG2_MAT_NU             YPVKKASYETLGKCAANKIPTVKKGVTPTPIQAYGGQVITYPVPVDAFEDVEDGGTRNLT
Hs_IG2_MAT_NU_6TIMES                  FKATSITVTGSGSC--NQRPELKNHI--DRVDAWVGTYFEVKIPSDTFYDHEDTTTDKLK
                                      : ... :    *.*  *: * :*: :    ::*: *  :   :* *:* * **  * :*.

Nv_6 copies of IG2_MAT_NU             LQLLSADGKELPKSYWLIFNSDTQTIRGLPGSRQVGVHNFIMEAKDSKGGKVRNPFKIDV
Hs_IG2_MAT_NU_6TIMES                  LTLKLREQQLVGEKSWVQFNSNSQLMYGLPDSSHVGKHEYFMHATDKGGLSAVDAFEIHV
                                      * *   : : : :. *: ***::* : ***.* :** *:::* *.*. * .. :.*:* *

Nv_6 copies of IG2_MAT_NU             GE----DTTPTSLLFTVYLDYDDDKFLNDVKVRLGLVDKIV-DYFKINENEMQVDRYGSG
Hs_IG2_MAT_NU_6TIMES                  HRRPQGDRAPAR--FKAKFVGDPALVLNDIHKKIALVKKLAFAFGDRNCSTITLQNITRG
                                            * :*:   *.. :  *   .***:: .:.**.*:.  : . * . : ::.   *

Nv_6 copies of IG2_MAT_NU             ------VNFTFHLKTIPEKDCNHPSRTTAINKFGRRGKTSSAFKNALLPEYNVRQSFTEA
Hs_IG2_MAT_NU_6TIMES                  SIVVEWTNNTLPLEPCPKEQIAGLSRRIAEDD----GKPRPAFSNALEPDFKATSITVTG
                                            .* *: *:. *:::     **  * :.    **. .**.*** *:::. .  . .
                                                         5X-------->
Nv_6 copies of IG2_MAT_NU             IGECSAKGNTPPLLLTPVGTIAVQANHGFIYRIPYDTFFDREYGNTRNLDLRLRAGDRSR
Hs_IG2_MAT_NU_6TIMES                  SGSCNQR----PELKNHIDRVDAWVGTYFEVKIPSDTFYDHEDTTTDKLKLTLKLREQQL
                                       *.*. .    * * . :. : ....  *  .** ***:*.*  .* :*.* *.  :..

Nv_6 copies of IG2_MAT_NU             LPPGSWVTLEEAEQEIYIMPDEKLTGSHKFVLVAVDPAD-NKMKTHDVITIKVSEPVPPT
Hs_IG2_MAT_NU_6TIMES                  VGEKSWVQFNSNSQLMYGLPDSSHVGKHEYFMHATDKGGLSAVDAFEIHVHRRPQGDRAP
                                      :   *** ::. .* :* :**.. .*.*::.: *.* .. . :.:.:: . . .:   ..

Nv_6 copies of IG2_MAT_NU             TIFSLLLDTDEKAFQGNVKTRMGLLTKLAEYFKVTIPDIHIISYKPEDPG-IRFTFGFSN
Hs_IG2_MAT_NU_6TIMES                  ARFKAKFVGDPALVLNDIHKKIALVKKLA--FAFGDRNCSTITLQNITRGSIVVEWTNNT
                                      : *.  :  *   . .:::..:.*:.*** *.   :   *: :    * * . :  ..

Nv_6 copies of IG2_MAT_NU             VPTKPCDSPALAKLIELFC--DSKVTDGLKDALKSCFTVKNGRVEQVGACKAPDINTAPK
Hs_IG2_MAT_NU_6TIMES                  LPLEPCPKEQIAGLSRRIAEDDGKPRPAFSNALEPDFKATSITVTGSGSC-----NQRPE
                                      :* :** .  :* *  :. *.*   .:.:**:..*....  *   *:*      *  *:
                                          6X-------->
Nv_6 copies of IG2_MAT_NU             LFNAFDRLNVFGTQGLRFDIPGDSFFDKEDLYTKNLTLTLKLGEGDLGSLLPTSWILLNS
Hs_IG2_MAT_NU_6TIMES                  LKNHIDRVDAWVGTYFEVKIPSDTFYDHEDTTTDKLKLTLKLREQQL--VGEKSWVQFNS
                                      * * :**::.:    : ..**.*:*:*:**  *.:*.***** * :*  :  .**: :**

Nv_6 copies of IG2_MAT_NU             TSQQIYALPMSDAIGLHKFVLEAADSGGLKAFDAFEVSVIKDEAG--FNHQFTIFLNFDN
Hs_IG2_MAT_NU_6TIMES                  NSQLMYGLPDSSHVGKHEYFMHATDKGGLSAVDAFEIHVHRRPQGDRAPARFKAKFVGDP
                                      .** :*.** *. :* *::.: *:*.***.*.****: * .   *     .*.  :  *

Nv_6 copies of IG2_MAT_NU             ATFMENVHVRLALVEKIAKFFG-VNFTAIRVDNAGLGPFMT-FHVAGIPLDDCNNPLLLK
Hs_IG2_MAT_NU_6TIMES                  ALVLNDIHKKIALVKKLAFAFGDRNCSTITLQNITRGSIVVEWTNNTLPLEPCPKEQIAG
                                      * .::::* .:***:*:*  **  * ::* ::*   *.::. :    :**: * :  :

Nv_6 copies of IG2_MAT_NU             MQENFGPDG--VSPALKAALAP-YPVAGGDYTKTKPC
Hs_IG2_MAT_NU_6TIMES                  LSRRIAEDDGKPRPAFSNALEPDFKATSITVTGSGSC
                                      :. .:. *.    **:. ** * : .:.   * : .*
```

Fig. S2: ***Nematostella vectensis* DG includes tandem repetitions of the IG2_MAT_NU module.** A large insertion, downstream of the mucin-like region, includes five tandem IG2_MAT_NU modules, making a total of six modules. A CLUSTALW alignment of this region with the IG2_MAT_NU module of human DG (represented 6 times) is shown. In each representation of the human IG2_MAT_NU region, the amino acid residues are shown in yellow for the IG2 domain, blue for the MAT region and red for the NU domain. The symbol '*' indicates an identical residue; ':' a conservative substitution and '.' a semi-conservative substitution.

Biology Open • Supplementary information

```
Hs              -----------MRMSVGLSLLLP---LSGRTFLLLLSVVMAQSHWPSEPSEAVRDWENQL
Lc              -------------SSDLPPPFIASSTMTATLPTSTIASSSMQASQPVAPSSALFT-DTSI
Oc              SSTSLLTATLSSKFSSNFSVFSPSPTVNETSSLVISTSAVGTSSLSLSPTSVSATFSESS
Aq              ---------MDSFSTSIFESPTPSILLPETSSSMIFEYPTPSILLPETSSSMIFEYPTPS
Em              ------------------------------------------------------------

Hs              EASMHSVLSDLHEAVPTVVGIPDGTAVVGRSFRVTIPTDLIASSGDIIKVSAAGKEALPS
Lc              TSALDQQASTPVVAASTSVPVLESSA-----------QPIVSTSAVVSSLSV--------
Oc              SFISDSFTSTFPTILSSTSFLTATST-----FSVFSPSPTVNETSSVTPVFST-------
Aq              ILLPETSSSMPFESPTPSILLPETSS--SMIFEYSTPSILLPETSS--------------
Em              --------------SPTSSPLPSPTS----------------------------------
                               ..   :   ::

Hs              WLHWDSQSHTLEGLPLDTDKGVHYISVSATRL---GANGSHIPQ-TSSVFSIEVYPEDHS
Lc              ------------SSAVDVIDDVISSSQTSLRI---PVETPVVPS-TTGTPDIQITSTTAQ
Oc              ------------SFPQLNSSSIVTTSTASSEISLSPVSTTAVPSVTNSSF----------
Aq              ------------SMPFESPTPSILLPETSSSM---IFESPTLSSLLPETSSVSFILESVS
Em              ------------SLP---------------------------------------------
                            . .

Hs              ELQSVRTASPDPGEVVSSACAADEPVTVLTVILDADLTKMTPKQRIDLLHRMRSFS----
Lc              IAETTTLPSLLPSSVLLDEVFTTEVPTASSSPMDFLLPETTRASSSLVVSTEVEQITTVA
Oc              ----ATVTSRFTSATSIETTSFPSISVFSTSSLPPSVSSTTPFENSSTVFSSISAT----
Aq              ELFESPSPTLFPDSSSSEIIFETPISSSSGTFESPTPSFIEPSSSSDIMFDDPTSILQTS
Em              ------------------------------------------------------------

Hs              ---------EVELHNMKLVPVVNNRLFDMSAFMAGPGNAKKVVENGALLSWKLGCSLNQN
Lc              PEITPVLTTAVTSSSMTRVPDVSTVVIDFSPFATATADSSSSA----LPTPVATSIPLL
Oc              ----SVFTVASSNFSFTISPVSTTTAVSPTSFSFEVSQTPTSVS-----AVRNTTSVSFN
Aq              SFISPI---ASPSEDFTPIPSPTDEFSSTPSFLLSSSDEITSSS-----EFESSLILLFS
Em              ------------------------------------------------------------

Hs              SVP----DIHGVEAPAREGAMSAQLGYPVVG-WHIANKKPPLPKRVRRQIHATPTPVTAI
Lc              DQS----IADGSGLDTSEVTSTTPLAIPSTIGFEFSTVDLFPPSAS-----AQPSTTEFI
Oc              ESDTQVFTVTSVFSTSASVSSSSTKVFPSNF-SSTGPSTSLVPSVSTTPVLSKTSSANFS
Aq              SSS----EIISELLPTSSIEEEPFTTVPSIGESFTAVTSIYISSTSTTNKFPSSSFGDLS
Em              ------------------------LSMPSII-----------------------------
                                           *

Hs              GPPTTAIQEPP-SRIVPTPTSPAIAPPTETMAPPVRDPVPGKPTVTIRTRGAIIQTPTLG
Lc              IPSTTAGLD-----IIP-------------------------------------------
Oc              VVISSTSLSPTLVPSLPVVNSTSSIGVSTSVAPNSTTVSSTTAVNTTLSSVSPVPTTSVQ
Aq              SSVVTESFSMDISSIVPSPPSTQTPTPSSTHGPPFASITSELFSSTSEIPFESSSEFITE
Em              ------------SSSLP-------------------------------------------
                               :*

Hs              PIQPTRVSEAGTTVPGQIRPTM-TIPGYVEPTAVATPPTTT---TKKPRVSTPKPATPST
Lc              PLLSSPHLSVFSTATEEVFTTIETQPASSTSVGMSTPLLSTDVGSGE--FTLPTPVSSSG
Oc              TSVSTPVPNVTTSAVFSTSSFLRSISPTPNVSTIQTPASSSVVATSSSFISTIVPKATST
Aq              SFFTPFVSESTSFQSPSIDMTRSLPPSESTSTIVSSPVTSSKITSGSEFSTFLPPSTEES
Em              ----------------------------------------------------PSPLTSST
                                                                      * : .
```

Biology Open • Supplementary information

```
Hs              DSTTTTTRRPTKKPRTPRP--------------VPRVTTKVSITRLETASP---------
Lc              PVLDASLSTVSVASTQDIS--------------TARLTTSELVSSFEFSSPMATASPM--
Oc              SVPPSSISTSPVTVTSIFS--------------NVSISLSSLVASISSSISVTSISSG--
Aq              MSLSASISPSSTLLSVIVSSFIEESSTSITLDVTSTITEPSFDSSFLFSTSLTKVTSLVE
Em              SSLSPT------------------------------------------------------
                    .:

Hs              --------PTRIRTTTSGVPRGGE-PNQRPELKNHIDRVDAWVGTYFEVKIPSDTFYDHE
Lc              -----TTVIVTTPVVTTAPPTPAP-VDNPPEINGDLQQYDVLVGEYLEMVLDPSHFIDED
Oc              -----TSSPTSVVPTPTPTPTPGP-PNTAPFVLNQIQRISVFANRPYFFLIPGDTFYDMQ
Aq              ISPSSTKIFSPTITSSSEAPSPSP-TNNRPLLLNPIGALLVQEGTVFSHSIPDDTFYDIE
Em              -------------PSTTPSPTPCPYGNKPPVAASPIGIVLVNEGQLFQFTISKTAFVATA
                               .:  *      :  *   . :    .  .      :    *

Hs              DTTTDK----LKLTLKLREQQLVGEKSWVQ-FNSN---------SQLMYGLPDSSHVGKH
Lc              TNGSLCAVCMLQLHLLNIVGNRVADSGPVRLANTTPSADGTFRRTLQIYGLPR--HGNSI
Oc              DGDTRA----LTLSLRTLDNSPPAADSWIQ-LRED---------SQELVMLPSFFELGST
Aq              DG--------YNLTLRLQS-----EVNWIGIMNQN---------AIIGLPLSTEIIGHYI
Em              PGN-------LTLTLLDTSGNALPNTSWVQ-LRGS---------TLEGVALRDAVVMGAI
                            * *           . :   .           :     *

Hs              E-YFM--HATDKGGLSAVDAFEIHVHRRPQGDRAPARFKAKFVGDPALVLNDIHKKIALV
Lc              TPYVL--RAMDSRGNVADDAFAVSVLPFPDGVPLSNEITVLLQENFTVFNSTWLRQVQFM
Oc              I-FLL--FATDKEGLIARMTFEVFVN-IPPIVTSTNVITQSFDVNFQAVNADTLQKLKLI
Aq              TRYNIEIRAYDRNGRFASEIITVSVNPR-HGLLLENYIQVSVDENFTTFNENVRAKVDLI
Em              TDLVLVVRASDDCGGSAYNFATIRVLPQPSRV---NFLLVHVEGDYLMASQSLSTRIRLV
                    :   * *  *  *     : *            :   .  :          .: ::

Hs              KKL--AFAFGDRNCSTITLQNITRGSIVVEWTNNTLPL-EPCPKEQIAGLSRRIAEDDGK
Lc              SSLQSAITATGSQPQLLMVFPIQQGSVIARVSDIALVS---C--------------DSGQ
Oc              DDL--ANFAKV-DPALVLVNDIREGSLVLSWSLTNITSIDDC--STVYKIFNSMF-NGDD
Aq              TKL------GMSDPNDIYVYSLTSGSVVISYTNISIRS-DDC--KEFNNFVNSIY-HSRN
Em              QRL--AAFGGSTNPRAIYVAEFREGTVGVLYANLSISD-FEC--EAFRSWTLSIY-VEGN
                  *         :   : :  :  *::    :   :     *                 .

Hs              PRPAFSNALEPDFKATSITVTGSGSCRHLQFIPVVPPRRVPSEAPPTEVPDRDPEKSSED
Lc              RQDQLNTASALRSLGTNATVN--VTCQS--VTVQEPTAEVGFAISPDT---------GQM
Oc              VTPEFAAALSEFKPQKSSSLT--GVCNNPIVVTQPPVVTVAGLNEGVE-----PDALSDS
Aq              YTDAFKTQINPYIPIGSLKVF--GTCNRSVGTYETIQPTIGIIDDPIS----------EQ
Em              YTWQFRDAILPFVAAKVPSFV--GPCS---VSSFNITPTLGSKATNIS-----SLATTET
                    :             ..     *             :                  :
                              TM
Hs              DVYLHTVIPAVVVAAILLIAGIIAMICYRKKRKGKLTLED---QATFIKKGVPIIFADEL
Lc              IDSLNVIIPVSAVLGVVLIAAVVATVYYRRHRPERKKLRGTRMRRTFDRRNPILISGERD
Oc              ESYVNFVVPAIVIGAVLLVVGVLVFMLYRARRKSRQDIEASRGTSTYFDRTPVVFGNEAT
Aq              LIFLAVVLPLVVLACILLVLCLVVFITYRWRRMERNYILN-EEQKILLNRKPIVFLDESE
Em              TSLLETIVPLVVVAFLLLAAGIAGCLLYRFHRPERKHLYG--SRSTYLNRQP-VYMNGEL
                   :  ::*  .:  ::*   :   : ** .*  .  :           .   :

Hs              DDSKPPPSSSMPLILQEEKAPLPPPEYPNQSVPETTPLNQDTMGEYTPLRDEDPNAPPYQ
Lc              RQRRRPRPPT----VLRSETRAPVGRRTTSVRLAELIEEGEMTSLMADLDEEGTR-PQFN
Oc              GDGIDGAQPAR---IVDTPSPPPFGQRGEPTDS--GEEGGDEISFHSGEGDAGSS-PSVL
Aq              SLSRSRRPTILAERLRGSGYHSLMNTESEGLDDTFTTIPEHDTVSLT---DEALQFPFVE
Em              NLPIRRRGPLF---IQGEGEPATLTRERAPVMGEHGYDHPDRTVDFSDEDDELVNVALLA
                        .     :                               :   :     .

Hs              PPPPFTAPMEGKGSRPKNMTPYRSPP----------------------------------
Lc              EPPPFH----------VAMHERRGPP----------------------------------
```

Biology Open • Supplementary information

```
Oc              PPPVQSSGRREAPTAKRAVPAYRKPP----------------------------------
Aq              ISGP---------SHPNALPPYRHPP----------------------------------
Em              ARQPVW-------QHASTTPPYRLPPVCVQKDGQLAQASTDYSGLLDGEDGGTIEMGPDT
                                      * **
Hs              ------------PYVPP------
Lc              -----------PAYTLPPDYILF
Oc              ------------PYSLPSDID--
Aq              ------------QLR--------
Em              SSEEEGGRHSSPEYRLPESARLL
```

Fig. S3: MUSCLE 3.8 multiple sequence alignment of poriferan dystroglycan-like proteins with human DG. Key: Hs, *Homo sapiens*; Lc, *Leucosolenia complicata*; Oc, *Oscarella carmela*; Aq, *Amphimedon queenslandica*; Em, *Ephydatia muelleri*. Key conserved residues are in red. The transmembrane domain is underlined. The symbol '*' indicates an identical residue; ':' a conservative substitution and '.' a semi-conservative substitution.

Biology Open • Supplementary information

Supplemental Table 1: GenBank or Compagen Accession codes of the proteins reported in Fig. 6B. Predicted laminin subunits in addition to those reported in this Table were identified in O. *carmela, H. magnipapillata, N. vectensis* and *A. digitifera*. Key: + probable partial sequence, signal peptide not identified.

Phylum/Species	α-Sarcoglycan	Sarcospan	Dystroglycan	Laminin-α	Laminin-β	Laminin-γ	Dystrophin	SGK196/ POMK	B4GAT1 (ex B3GNT1)	LARGE1
**Filastereans**										
C. owczarzaki							CR only: XP_004363347/ GI:470306037	XP_004342773/ GI:470289127	XP_004346787/ GI:470300042	XP_004365036/ GI:470361182
**Choanoflagellates**										
M. brevicollis MX1							CR only: XP_001742662/ GI:167516642		XP_001747298/ GI:167525928	XP_001742373/ GI:167516064
S. rosetta							CR only: XP_004994334/ GI:514693524	XP_004994181/ GI:514693218	XP_004991716/ GI:514688280	XP_004993018/ GI:514690889
**Ctenophores**										
M. leidyi				ML097515a(MGP)+		MLRB05952 (MGP) and ML21866a(MGP) (β/γ-like)+				
P. bachei										
**Porifera**										
A. queenslandica				XP_003382371/ GI:340367659	XP_003387987/ GI:340378944	XP_003390574/ and XP_003386652	XP_011403729.1 GI:761908478			
O. carmela		g5110.t1(GPEP-COMPAGEN)	comp34447 (TPEP-COMPAGEN) (IG2→DBS)+	g7319.t1(GPEP-COMPAGEN)		g7968.t1 (GPEP-COMPAGEN)	g991.t1(GPEP-COMPAGEN)	g10651.t1(GPEP-COMPAGEN)		g4929.t1(GPEP-COMPAGEN)
**Placozoa**										
T. adhaerens			XP_002116019/ GI:196012313	XP_002114273/ GI:196008815 (incomplete sequence+)	XP_002111840/ GI:196003946	XP_002109259/ GI:195998782	XP_002116093/ GI:196012461	XP_002117488/ GI:196015261	XP_002115499/ GI:196011271	XP_002117599/ GI:196015485
**Cnidaria**										
H. magnipapillata	Hma2.216143 (GPEP-COMPAGEN)	XP_002163153/ GI:221119018	XP_002164217/ GI:449662868	XP_002170373 /GI:449677846	XP_002168125/ GI:449666107	XP_002165286/ GI:449674332	XP_002162937/ GI:449686663	XP_004208080/ GI:449673996		XP_002154954/ GI:449666871
N. vectensis	XP_001641924 /GI:156408560		XP_001629936/ GI:156375132	Nemve1-209260	XP_001631565/ GI:156379641	XP_001628586/ GI:156371064	XP_001633160/ GI:156384081	XP_001640835/ GI:156405631	XP_001627257/ GI:156367099	XP_001640724/ GI:156405409
A. digitifera	adi_v1.06362 (GPEP-COMPAGEN)		adi_v1.10683 (GPEP-COMPAGEN)	adi_v1.10227 (GPEP-COMPAGEN)	adi_v1.10769 (GPEP-COMPAGEN	adi_V1.06175 (GPEP-COMPAGEN)	adi_v1.08652 (GPEP-COMPAGEN)	adi_v1.14395 (GPEP-COMPAGEN)		adi_v1.15902 (GPEP-COMPAGEN)

Biology Open • Supplementary information
